# Evaluating the performance of five large language models in answering Delphi consensus questions relating to patellar instability and medial patellofemoral ligament reconstruction

**DOI:** 10.1186/s12891-025-09227-1

**Published:** 2025-11-03

**Authors:** Prushoth Vivekanantha, Dan Cohen, David Slawaska-Eng, Kanto Nagai, Magdalena Tarchala, Bogdan Matache, Laurie Hiemstra, Robert Longstaffe, Bryson Lesniak, Amit Meena, Sachin Tapasvi, Petri Sillanpäa, Patrick Grzela, Daniel Lamanna, Kristian Samuelsson, Darren de SA

**Affiliations:** 1 Department of Surgery, Division of Orthopaedic Surgery, McMaster University, 1200 Main St West, Hamilton, ON L8N 1H4 Canada; 2 Department of Orthopaedic Surgery, Kobe University Graduate School of Medicine, Kobe, Hyogo Japan; 3 Department of Surgery, Division of Orthopaedic Surgery, University of Ottawa, Ottawa, ON Canada; 4 Department of Surgery, Section of Orthopaedic Surgery, University of Calgary, Calgary, AB Canada; 5 Department of Surgery, Section of Orthopaedic Surgery, University of Manitoba, Winnipeg, MB Canada; 6 Department of Orthopaedic Surgery, University of Pittsburgh Medical Center, Pittsburgh, PA USA; 7 Department of Orthopaedics and Trauma, Shalby Hospital Jaipur, Jaipur, India; 8 The Orthopaedic Specialty Clinic, Pune, Maharashtra India; 9 Pihlajalinna Hospital, Tampere, Finland; 10 Department of Orthopaedics, Sahlgrenska University Hospital, Mölndal, Sweden; 11 Department of Orthopaedics, Institute of Clinical Sciences, the Sahlgrenska Academy, The University of Gothenburg, Gothenburg, Sweden

**Keywords:** Large language models, Chatbots, Artificial intelligence, Patellar instability, Medial patellofemoral ligament reconstruction

## Abstract

**Purpose:**

Artificial intelligence (AI) has become incredibly popular over the past several years, with large language models (LLMs) offering the possibility of revolutionizing the way healthcare information is shared with patients. However, to prevent the spread of misinformation, analyzing the accuracy of answers from these LLMs is essential. This study will aim to assess the accuracy of five freely accessible chatbots by specifically evaluating their responses to questions about patellofemoral instability (PFI). The secondary objective will be to compare the different chatbots, to distinguish which LLM offers the most accurate set of responses.

**Methods:**

Ten questions were selected from a previously published international Delphi Consensus study pertaining to patellar instability, and posed to ChatGPT4o, Perplexity AI, Bing CoPilot, Claude2, and Google Gemini. Responses were assessed for accuracy using the validated Mika score by eight Orthopedic surgeons who have completed fellowship training in sports-medicine. Median responses amongst the eight reviewers for each question were compared using the Kruskal-Wallis and Dunn’s post-hoc tests. Percentages of each Mika score distribution were compared using Pearson’s chi-square test. P-values less than or equal to 0.05 were considered significant. The Gwet’s AC2 coefficient was calculated to assess for inter-rater agreement, corrected for chance and employing quadratic weights.

**Results:**

ChatGPT4o and Claude2 had the highest percentage of reviews (38/80, 47.5%) considered to be an “excellent response not requiring classification”, or a Mika score of 1. Google Gemini had the highest percentage of reviews (17/80, 21.3%) considered to be “unsatisfactory requiring substantial clarification”, or a Mika score of 4 (*p* < 0.001). The median ± interquartile range (IQR) Mika scores was 2 (1) for ChatGPT4o and Perplexity AI, 2 (2) for Bing CoPilot and Claude2, and 3 (2) for Google Gemini. Median responses were not significantly different between ChatGPT4o, Perplexity AI, Bing CoPilot, and Claude2, however all four statistically outperformed Google Gemini (*p* < 0.05). Inter-rater agreement was classified as moderate (0.40 > AC2 ≥ 0.60) for ChatGPT, Perplexity AI, Bing CoPilot, and Claude2, while there was no agreement for Google Gemini (AC2 < 0).

**Conclusion:**

Current free access LLMs (ChatGPT4o, Perplexity AI, Bing CoPilot, and Claude2) predominantly provide satisfactory responses requiring minimal clarification to standardized questions relating to patellar instability. Google Gemini statistically underperformed in accuracy relative to the other four LLMs, with most answers requiring moderate clarification. Furthermore, inter-rater agreement was moderate for all LLMs apart from Google Gemini, which had no agreement. These findings advocate for the utility of existing LLMs in serving as an adjunct to physicians and surgeons in providing patients information pertaining to patellar instability.

**Level of evidence: V:**

**Supplementary Information:**

The online version contains supplementary material available at 10.1186/s12891-025-09227-1.

## Introduction

Artificial intelligence (AI) has become increasingly popular over the past few years [1]. One of the most mainstream usages of AI is in the form of large language models (LLMs), such as ChatGPT (Open AI, San Francisco, United States) or Perplexity (Perplexity AI, San Francisco, United States) [2, 3]. These LLMs offer novel opportunities to enhance patient education and care [4–6]. Recent studies have investigated the quality of responses from LLMs, notably ChatGPT, to various questions pertaining to orthopedic pathologies and surgeries. This includes anterior cruciate ligament reconstruction (ACLR), hip arthroscopy, and ulnar collateral ligament reconstruction (UCLR), generally finding satisfactory responses when reviewed by experts [7–9].

Patellofemoral instability (PFI) is a common orthopedic condition that may require surgical intervention in the form of medial patellofemoral ligament reconstruction (MPFLR) [10, 11]. It has an incidence between 21.6 and 49.7 per 100 000 persons [12]. PFI has been shown to cause similar levels of dysfunction to the knee as that of an ACL tear [13]. It is the responsibility of the healthcare team to ensure that patients receive accurate, digestible, and up-to-date information about their condition, and what may be required from a treatment and recovery point of view. However, LLMs may serve as a useful adjunct, where patients would be able to have immediate access to information outside of hospital and clinic visits. Given this, assessing the use of LLMs responses for pathologies such as PFI is essential to ensure patient safety.

This study will aim to assess the accuracy of five freely accessible chatbots: ChatGPT4o, Perplexity AI, Claude2 (Anthropic, San Francisco, United States), Microsoft Copilot (Microsoft, Redmond, Washington), and Gemini (Google DeepMind, London, United Kingdom), by specifically evaluating their responses to questions about PFI. The secondary objective will be to compare the different chatbots, to distinguish which LLM offers the most accurate set of responses.

## Materials and methods

### Question selection

Questions that were selected were based upon a previous modified Delphi Consensus statement study on patellar instability [13, 14]. This consensus statement was developed in collaboration with 60 surgeons, all either members of one or more of the following societies: American Orthopedic Society for Sports Medicine (AOSSM), Arthroscopy Association of North America (AANA), European Society of Sports Traumatology, Knee Surgery and Arthroscopy, International Society of Arthroscopy (ESSKA), Knee Surgery and Sports Medicine (ISAKOS), and the Patellofemoral Foundation [13, 14]. Questions were only chosen if there was unanimous consensus or if there was strong consensus, as per the original manuscript. The questions were as follows:

1. What factors of patient history should be evaluated in the setting of patellar instability?
2. What aspects of the physical exam should be performed in patients with patellar instability?
3. When should advanced imaging (MRI/CT) be performed in patients presenting with patellar instability?
4. When should patients start range of motion exercises when undergoing non-operative management for patellar instability?
5. What are the indications for nonoperative management for patients with patellar instability?
6. What are the contraindications for nonoperative management for patients with patellar instability?
7. What are the indications for MPFL reconstruction for patients with patellar instability?
8. Are there any different considerations that should be made in pediatric patients undergoing MPFL reconstruction for patellar instability?
9. What clinical factors may influence your decision to perform a TTO for patellar instability?
10. Which factor(s), if any, should be considered when deciding which trochleoplasty technique to perform?

### Accuracy assessment of responses

The ten questions were inputted into ChatGPT4o, Perplexity AI, Claude2, Microsoft Copilot, and Google Gemini in August 2024. There were no prior prompts used nor were there any repeat or follow-up questions asked. Answers were assessed by eight orthopedic surgeons with fellowship training in sports medicine using the rating system described by Mika et al. [15, 16]. This grading system has been used extensively in the past by a variety of studies with similar aims [8, 17, 18]. This system consists of four categories: Response accuracy was rated as (1) “excellent response not requiring clarification” if it provided fundamentally factual information free of inaccuracies; (2) “satisfactory requiring minimal clarification” if it provided correct information but was missing some finer points or nuances; (3) “satisfactory requiring moderate clarification” if it provided outdated information or information that was not relevant to the question asked; or (4) “unsatisfactory requiring substantial clarification” if it provided incorrect or overly generalized information that could be conceivably misinterpreted or detrimental [15, 16]. Surgeons were not blinded during this study.

### Statistical analysis

Descriptive statistics, such as medians, interquartile ranges (IQR), means, ranges, and percentages were utilized. Median scores for each LLM were calculated, compared using the nonparametric Kruskal-Wallis and Dunn’s post-hoc tests. A two-tailed Pearson’s chi-square test was conducted comparing percentages of Mika scores, with adjusted standardized residuals used to identify where significant differences occurred. Values greater than positive or negative 2 were considered percentages differing significantly from expectation. P-values less than or equal to 0.05 were considered to be statistically significant. Gwet’s AC2 coefficient employing quadratic weights was utilized for assessment of agreement. This coefficient accommodates weighted values for ordinal variables, making it suitable for comparing Mika score values. The Landis and Koch categorization was adopted as per previous studies, given the lack of well-established thresholds for Gwet’s AC2 coefficient [19–21]. The categorization of the AC2 coefficient was defined a priori as: 1.00 >AC2 ≥ 0.80 indicates almost perfect agreement, 0.80 >AC2 ≥ 0.60 indicates substantial agreement, 0.60 >AC2 ≥ 0.40 indicates moderate agreement, 0.40 >AC2 ≥ 0.60 indicates fair agreement, 0.20 >AC2 ≥ 0.00 indicates slight agreement and a AC2 score = 0 indicates no agreement [20]. For each Gwet’s AC2 coefficient, 95% confidence intervals (CI) were also calculated. All statistics were performed using Excel (Microsoft, Redmond, Washington, USA) or Python (version 3.11; Python Software Foundation, Wilmington, Delaware, USA).

## Results

### Accuracy of LLM responses

**Fig. 1:**
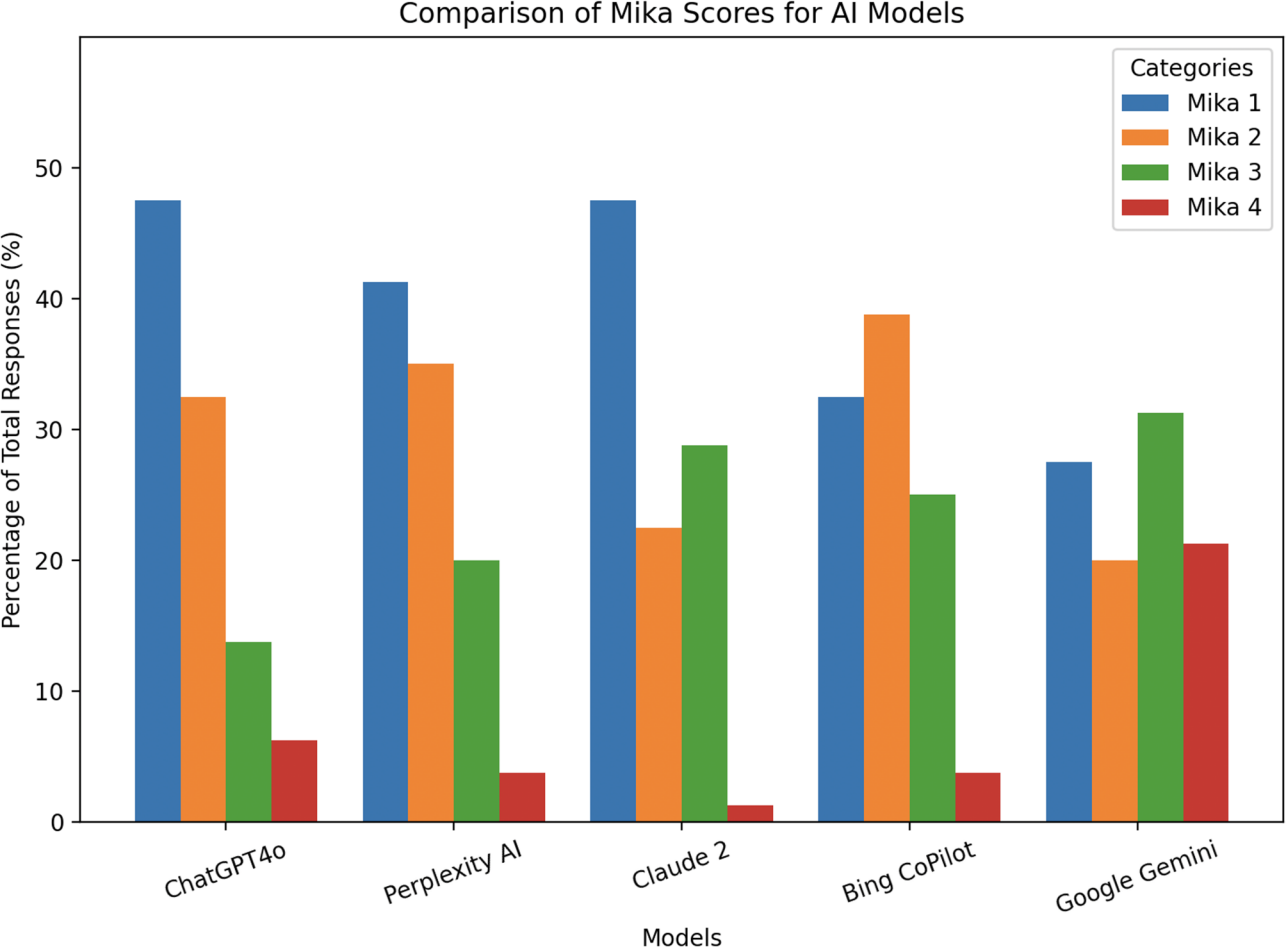
Number of responses per large language model (LLM) categorized by the Mika score. Accuracy scores were defined as (1) “excellent response not requiring clarification”; (2) “satisfactory requiring minimal clarification”; (3) “satisfactory requiring moderate clarification”; or (4) “unsatisfactory requiring substantial clarification”

The median (IQR) Mika score for ChatGPT4o and Perplexity AI was 2 (1) for both, while the median (IQR) Mika score for Bing CoPilot and Claude2 was 2 (2) for both. The median (IQR) Mika score for Google Gemini was 3 (2) (Table 1). Amongst 80 reviews to responses, ChatGPT4o and Claude2 had the highest amount rated as a Mika 1 (*n* = 38; 47.5%), followed by Perplexity AI (*n* = 33; 41.3%). Google Gemini had 31.3% (*n* = 25) and 21.3% (*n* = 17) rated as a Mika 3 and 4, respectively (Fig. 1). Statistical comparisons using the Kruskal-Wallis test found a significant difference between LLMs (*p* = 0.0005). Dunn’s post-hoc tests found that ChatGPT (*p* = 0.00002), Perplexity AI (*p* = 0.002), Bing Copilot (*p* = 0.01), Claude2 (*p* = 0.0002) all outperformed Google Gemini in accuracy. No significant differences were found between ChatGPT, Perplexity AI, Bing CoPilot, and Claude2. Statistical comparisons of distribution of Mika scores across the fives LLMs using the Pearson’s chi-square test was also significant (X^2^ = 48.74, *p* < 0.001). Using adjusted standardized residuals, Google Gemini was found to have significantly fewer Mika scores of 1 (adjusted residual = −2.41) and 2 (adjusted residual = −2.13), but more Mika scores of 4 (adjusted residual = + 5.40). Claude2 was found to have significantly fewer Mika scores of 4 (adjusted residual = −2.31). Additionally, ChatGPT4o had significantly fewer Mika scores of 3 (adjusted residual = −2.35) while Bing CoPilot had significantly more scores of 2 (adjusted residual = + 1.97).

**Table 1:** Individual reviewer responses to answers by large language models (LLMs)

ChatGPT4o								
Question#	Reviewer 1	Reviewer 2	Reviewer 3	Reviewer 4	Reviewer 5	Reviewer 6	Reviewer 7	Reviewer 8
1	1	1	2	2	3	2	1	1
2	2	2	2	3	3	1	2	1
3	2	1	1	3	3	1	1	1
4	3	1	2	3	4	1	2	1
5	3	2	1	4	1	1	1	1
6	2	2	1	2	1	1	1	1
7	4	2	1	2	2	1	2	1
8	3	1	2	2	2	1	1	1
9	3	1	2	4	2	1	2	1
10	3	1	2	4	2	1	1	1
Perplexity AI								
Question #	Reviewer 1	Reviewer 2	Reviewer 3	Reviewer 4	Reviewer 5	Reviewer 6	Reviewer 7	Reviewer 8
1	3	3	2	1	3	2	3	1
2	3	1	2	1	2	1	2	1
3	1	2	2	1	4	4	3	1
4	2	1	2	1	4	1	2	1
5	2	2	2	1	2	1	2	1
6	2	1	2	2	3	1	2	1
7	2	1	3	2	2	1	3	1
8	2	1	2	2	3	1	3	1
9	1	1	2	2	3	1	3	1
10	3	1	2	1	3	1	3	1
Bing CoPilot								
Question #	Reviewer 1	Reviewer 2	Reviewer 3	Reviewer 4	Reviewer 5	Reviewer 6	Reviewer 7	Reviewer 8
1	3	2	2	2	2	2	2	1
2	3	2	3	2	2	1	2	1
3	2	1	2	2	3	1	3	1
4	1	1	1	2	4	1	3	1
5	3	2	2	1	2	1	3	1
6	3	4	3	2	1	2	3	1
7	2	3	3	2	3	1	2	1
8	3	2	2	2	3	1	4	1
9	2	1	3	2	3	1	3	1
10	2	1	2	2	3	1	2	1
Claude2								
Question #	Reviewer 1	Reviewer 2	Reviewer 3	Reviewer 4	Reviewer 5	Reviewer 6	Reviewer 7	Reviewer 8
1	1	1	1	1	3	1	2	1
2	2	1	1	1	3	1	3	1
3	3	1	1	1	3	1	3	1
4	1	1	2	3	4	1	3	1
5	3	2	2	1	2	1	2	1
6	2	1	2	3	2	1	3	1
7	3	1	2	3	2	1	2	1
8	3	2	3	3	2	1	2	1
9	1	1	3	3	3	1	2	1
10	2	1	3	3	3	1	3	1
Google Gemini								
Question #	Reviewer 1	Reviewer 2	Reviewer 3	Reviewer 4	Reviewer 5	Reviewer 6	Reviewer 7	Reviewer 8
1	3	2	3	3	2	1	1	1
2	4	4	3	3	1	1	3	1
3	4	3	3	4	2	1	2	1
4	2	1	3	4	4	1	3	1
5	3	4	3	4	1	2	3	1
6	4	3	3	2	2	2	3	1
7	4	3	3	3	2	1	3	1
8	4	4	3	3	2	3	2	1
9	4	4	3	4	1	4	1	1
10	2	2	3	4	2	1	2	1

All chatbots had at least one response that was rated as “unsatisfactory requiring substantial clarification”, or a Mika score of 4, with all having at least one rating of 4 for question 4 (*“When should patients start range of motion exercises when undergoing non-operative management for patellar instability?”*). For ChatGPT4o, there were five total responses rated as a 4, however they were all for different questions (#4, #5, #7, #9, #10), with three (60%) from one reviewer. For Perplexity AI, there were three total responses rated as a 4, for two different questions (#3, #4) amongst two different reviewers. Bing CoPilot also had three total responses rated as a 4, amongst three different questions (#4, #6, #8) and three different reviewers. Claude2 only had one response rated as a 4 (#4). Five reviewers rated at least one response as a 4 for Google Gemini amongst nine of 10 total questions (all but #1). Full answers from each LLM are described in Supplementary Digital Material Tables 1, 2, 3, 4 and 5.

### Inter-rater agreement

Responses to four LLMs had moderate agreement, including ChatGPT (AC2 = 0.435; 95%CI 0.421, 0.44), Perplexity AI (AC2 = 0.450; 95%CI 0.400, 0.500), Bing CoPilot (AC2 = 0.435; 95%CI 0.395, 0.476), and Claude2 (AC2 = 0.457; 95%CI 0.434, 0.481). There was no agreement for Google Gemini (AC2 = −0.039; 95%CI −0.076, −0.002).

## Discussion

The primary finding of this study was that ChatGPT4o, Perplexity AI, Bing CoPilot, Claude2 generally provided “satisfactory responses requiring minimal clarification” to standardized questions relating to patellar instability and MPFLR. Google Gemini underperformed relative to these four LLMs, generally generating “satisfactory responses requiring moderate clarification”, with a large portion of responses considered to be “unsatisfactory requiring substantial clarification”. Responses from all LLMs apart from Google Gemini had moderate agreement amongst reviewers, indicating heterogeneity in the perceptions of responses from different chatbots.

Over the past few years, there have been several different LLMs that have been created by various companies, each improving on a yearly basis in performance capabilities [22]. Despite this, research focused on assessing the accuracy of LLMs in answering medical questions consistently only evaluates ChatGPT [22]. This is likely due ChatGPT being the most mainstream version of all the LLMs [22]. This study suggests that for the purposes of answering questions related to patellar instability, Google Gemini underperforms compared to ChatGPT4o, Perplexity AI, Bing CoPilot, and Claude2, however findings should be interpreted with caution due to poor inter-rater reliability. There have been a variety of studies in various other disciplines such as ophthalmology and emergency medicine which have demonstrated that Gemini was outperformed by ChatGPT-4 [23–27]. However other studies such as that by Quinn et al. have demonstrated superiority with Gemini with regards to clarity of answers pertaining to ACLR [28]. Overall, as AI models are fundamentally free of judgment, patients may feel comfortable asking questions that they may perceive to be “unintelligent”, facilitating clinic visits [29]. Therefore, ChatGPT4o, Perplexity AI, Bing CoPilot, and Claude2 are satisfactory adjuncts to surgeons in providing information regarding patellar instability and MPFLR, and are encouraged to be used by patients with this condition.

In addition to accuracy, inter-rater agreement is also an important metric when assessing the different models. Agreement between reviewers generally was over 0.400 using the Gwet AC2 coefficient in this study. In comparison, a similar study investigating the accuracy of ChatGPT in answering questions related to ACLR found an intra-class coefficient of 0.802 [8]. Another analysis found more similar results to the present study, inter-rater reliability values ranging from 0.28 to 0.43 for assessing the responses of ChatGPT for reliability, relevance, and accuracy in answering questions relating to total knee arthroplasty [30]. It is inevitable that reliability values will vary from study to study, even if they are investigating the same topic, given the different reviewers that assess the responses. It is unclear why Google Gemini had a low inter-rater reliability score, but it may be due to three reviewers scoring most of the responses from this chatbot as either a Mika 1 or 2. When these two are removed from analysis for Google Gemini, the Gwet AC2 coefficient increases to 0.509 (95%CI 0.420–0.598).

However, patellar instability and MPFLR is still a field that is rapidly evolving [13]. The Delphi Consensus study that the questions for this study were derived from concluded that more high-level evidence is necessary for development of universal standards [13]. While the ten prompts from this study were created from questions that had a strong consensus [13], there is still ongoing discourse about these aspects of patellar instability. For example, regarding the indications for operative versus nonoperative management, one recent systematic review reported a significant benefit in lowering redislocation rates with MPFLR for acute first-time patellar dislocation in the pediatric population (3.1% vs. 39.4%) [10]. Another systematic review reported a lower dislocation rate in patients with MPFLR compared to nonoperative management (7% vs. 30%) [11]. Other examples of controversy in this field include the use of isolated MPFLR for all patients (e.g. including patients with elevated tibial tubercle to trochlear groove (TT-TG)) distances [31], the use of tibial tubercle to posterior cruciate ligament (TT-PCL) distances versus TT-TG [32], measurement modality of TT-TG (e.g. MRI versus CT) [33], and an updated classification for trochlear dysplasia [34]. While the reasons for disagreement between quality of responses are multifactorial, the lack of definitive answers for various aspects of patellar instability in light of a lack of high quality evidence is likely a major contributor.

Several studies in general have described that LLMs provide satisfactory responses to questions related to orthopedics [8, 9, 35]. It can be concluded that for most orthopedic conditions, these LLMs are useful adjuncts to physicians and surgeons, and that patients should use them to gain information. As these softwares continue to develop, the potential for its usage will exponentially grow. The latest version of ChatGPT from OpenAI is now able to handle text, speech, and visual inputs, while also generating text, audio, and visual outputs. Using these new features to facilitate day-to-day workflow, analyze outcomes, streamline research, and enhance patient satisfaction can be the goal of future research in the AI space. In the context of PFI, future research should investigate the development of tailored chatbots trained on specific rehabilitation and postoperative protocols to provide reliable resources for patients.

This study is amongst few to assess the accuracy of five different LLMs within orthopedic research and patellar instability. Furthermore, all responses were assessed by a high number of practicing orthopedic surgeons, who all completed fellowship training within sports medicine. The questions chosen to prompt the LLMs were all from a previous Delphi Consensus study, all with strong consensus. Finally, all LLMs included in this study were free access, increasing generalizability. However, this study does have few limitations. First, surgeons were not blinded when reviewing responses from different LLMs, which may introduce bias. Second, despite moderate agreement as per Gwet’s AC2 coefficient for most LLLMs, having humans assess the accuracy of responses introduces a level of bias within grading. Third, LLMs are updated frequently, and the quality of responses are likely to improve with each subsequent update. Finally, this study only evaluated 10 questions related to PFI, limiting the ability to assess a broader and more diverse range of issues across this field. Capturing this phenomenon is not possible with a cross-sectional design.

## Conclusion

Current free access LLMs (ChatGPT4o, Perplexity AI, Bing CoPilot, and Claude2) predominantly provide satisfactory responses requiring minimal clarification to standardized questions relating to patellar instability and MPFLR. Google Gemini statistically underperformed in accuracy relative to the other four LLMs, with most answers requiring moderate clarification. Furthermore, inter-rater agreement was moderate for all LLMs apart from Google Gemini. These findings advocate for the utility of existing LLMs in serving as an adjunct to physicians and surgeons in providing patients information pertaining to patellar instability and MPFLR.

## Supplementary Information

Supplementary Material 1

## Data Availability

Data may be made available upon reasonable request.
