# Supplementary material for: Evaluating the performance of five large language models in answering Delphi consensus questions relating to patellar instability and medial patellofemoral ligament reconstruction
Source: BMC Musculoskelet Disord. 2025 Nov 3;26:1022. doi: 10.1186/s12891-025-09227-1 (PMC12581441; doi:10.1186/s12891-025-09227-1)
Supplement: Supplementary file 1 — Supplementary Material 1 [file 12891_2025_9227_MOESM1_ESM.docx]

**Table 1.** ChatGPT4o

| **Question** | **Answer** |
| --- | --- |
| **What factors of patient history should be evaluated in the setting of patellar instability** | When evaluating a patient with patellar instability, several factors from their history should be considered to get a comprehensive understanding of their condition. Key aspects to assess include:   1. History of Patellar Dislocations or Subluxations:    - Frequency and severity of episodes.    - Whether the dislocations were traumatic or occurred with minimal force.    - Duration of dislocations and any need for reduction or medical intervention. 2. Previous Injuries or Surgeries:    - Any prior knee injuries or surgeries, especially those involving the patella or surrounding structures.    - Previous treatments or interventions for patellar instability. 3. Activity Level and Sports Participation:    - Involvement in sports or activities that put stress on the knee, such as running, jumping, or twisting.    - Any specific movements or activities that seem to trigger instability or pain. 4. Pain and Functional Limitations:    - Description of the pain (e.g., sharp, dull, aching) and its location.    - Impact of the condition on daily activities and overall function.    - Any episodes of locking or catching in the knee. 5. Family History:    - Any family history of patellar instability, joint disorders, or genetic conditions that might predispose to instability. 6. Previous Treatments and Outcomes:    - Any conservative treatments tried, such as physical therapy, braces, or medications.    - Effectiveness of these treatments and any associated outcomes. 7. Mechanism of Injury:    - Details about the circumstances surrounding the initial dislocation or instability episode, including the type of trauma or force applied. 8. Associated Symptoms:    - Presence of swelling, bruising, or other signs of injury.    - Any concurrent symptoms like weakness, stiffness, or clicking. 9. Patient’s Overall Health:    - General health and any comorbid conditions that might affect treatment or recovery. 10. Alignment and Biomechanics:     - Any known issues with knee alignment or gait abnormalities.   Gathering this information will help in formulating a diagnosis, assessing the severity of the instability, and determining the most appropriate treatment plan. |
| **What aspects of the physical exam should be performed in patients with patellar instability** | In patients with patellar instability, a comprehensive physical examination is crucial for assessing the extent of the instability, identifying any contributing factors, and planning appropriate management. Here are the key aspects of the physical exam:   1. Inspection:    - Swelling and Bruising: Check for signs of swelling, bruising, or any deformity around the knee.    - Patellar Position: Observe the position of the patella in relation to the femur, noting any signs of misalignment or abnormal position. 2. Palpation:    - Patella: Palpate the patella for tenderness, crepitus, or abnormal movement.    - Medial and Lateral Structures: Palpate the medial and lateral retinaculum, tibial tuberosity, and other soft tissue structures around the knee to assess for tenderness or abnormalities. 3. Range of Motion:    - Active and Passive Range of Motion: Assess both active and passive range of motion of the knee to determine if there are limitations or pain associated with movement. 4. Patellar Tracking:    - Patellar Glide Test: Evaluate patellar tracking by moving the patella medially and laterally to see if it tracks properly within the trochlear groove. 5. Stability Testing:    - Lateral and Medial Patellar Stability: Assess the stability of the patella by applying gentle pressure to the patella in various directions while the patient is in different positions (e.g., supine, sitting).    - Patellar Apprehension Test: Test for patellar apprehension by applying lateral force to the patella while the patient is supine. A positive test is indicated by discomfort or the patient’s apprehensive response. 6. Strength and Muscle Function:    - Quadriceps Strength: Evaluate the strength of the quadriceps muscle, as weakness can contribute to patellar instability.    - Hip and Knee Musculature: Assess the strength and function of surrounding muscles, including hip abductors, adductors, and hamstrings. 7. Special Tests:    - McConnell Test: This test involves assessing patellar tracking with the patient in a seated position and then performing knee extension. It can help evaluate how patellar alignment affects symptoms.    - J-sign: Observe the patella during active knee extension to check for any abnormal lateral movement or tracking. 8. Functional Testing:    - Single-Leg Squats or Step-Ups: Perform functional tests to assess how the knee performs under load and evaluate for symptoms or instability during these activities. 9. Gait Analysis:    - Observe Gait: Assess the patient's gait for any abnormalities or compensatory movements that might indicate patellar instability or associated problems.   These aspects of the physical exam help to diagnose patellar instability, identify potential contributing factors, and guide the development of an appropriate treatment plan. |
| **When should advanced imaging (MRI/CT) be performed in patients presenting with patellar instability?** | Advanced imaging, such as MRI or CT scans, should be considered in patients with patellar instability under the following circumstances:   1. Uncertain Diagnosis or Severe Symptoms:    - When the clinical examination and history suggest patellar instability, but there is uncertainty about the extent of the injury or the exact pathology.    - If the patient has severe symptoms that are not responding to conservative management, advanced imaging can help clarify the diagnosis. 2. Recurrent Dislocations or Subluxations:    - In cases of recurrent patellar dislocations or subluxations, imaging can provide insight into underlying structural issues that might contribute to the instability. 3. Suspected Structural Damage:    - If there is a suspicion of associated structural damage, such as cartilage lesions, osteochondral injuries, or significant bone abnormalities.    - MRI is particularly useful for assessing soft tissue structures, including cartilage, ligaments, and the patellar retinaculum. 4. Assessment of Bone and Cartilage:    - When evaluating the patellar alignment, tracking, or the presence of bony abnormalities like a patellar fracture or malalignment.    - CT scans are often used to assess bony structures in more detail, particularly for evaluating patellar or trochlear dysplasia. 5. Preoperative Planning:    - When surgical intervention is being considered, advanced imaging helps in preoperative planning by providing detailed anatomical information. This can guide the choice of surgical technique and predict potential outcomes. 6. Evaluation of Failed Conservative Treatment:    - If conservative treatments such as physical therapy, bracing, or medications have failed, advanced imaging can help in reassessing the underlying pathology and guiding further treatment options. 7. Complicated Cases:    - In cases where there is a complex presentation with multiple contributing factors or associated injuries, advanced imaging can offer a comprehensive view of the knee’s condition.   MRI is generally preferred for evaluating soft tissue structures and assessing the extent of damage to cartilage, ligaments, and the surrounding soft tissues. It provides detailed images of the patella, retinaculum, and other soft tissue components.  CT scans are more useful for evaluating bony structures, including the alignment of the patella and trochlear groove, as well as detecting any bone lesions or abnormalities that might contribute to instability.  In summary, advanced imaging is indicated when there is a need to further investigate the extent of injury, assess for associated damage, or plan for surgical intervention. The decision should be guided by the clinical presentation, the results of the physical examination, and the response to initial conservative treatments |
| **When should patients start range of motion exercises when undergoing non-operative management for patellar instability?** | Range of motion (ROM) exercises are an important component of non-operative management for patellar instability. The timing for initiating these exercises should be based on several factors to ensure they are both safe and effective:   1. Acute Phase and Pain Management:    - Immediately Post-Injury or Surgery: In the acute phase, especially if there has been a recent dislocation or if the patient is experiencing significant pain and swelling, ROM exercises should be approached with caution. Focus initially on reducing pain and swelling through rest, ice, compression, and elevation (RICE), and possibly nonsteroidal anti-inflammatory drugs (NSAIDs) if appropriate. 2. After Initial Healing:    - Subacute Phase: Once the acute symptoms have improved (typically within a few days to a week), and the patient’s pain and swelling are under control, gentle ROM exercises can be introduced. The goal is to gradually restore normal knee movement without causing additional stress or pain. 3. Assessment of Stability and Function:    - Clinical Assessment: Begin ROM exercises when the knee is stable enough and the risk of causing further dislocation or injury is minimized. This assessment is usually done by a healthcare professional who will evaluate the patient’s knee stability and pain level. 4. Progression of Exercises:    - Controlled Motion: Start with gentle, pain-free ROM exercises to ensure that the patella moves within a safe range. Exercises such as heel slides, quad sets, and gentle knee flexion and extension can help improve mobility without overstressing the joint.    - Progressive Range: Gradually increase the range and intensity of exercises as tolerated. Include stretching and strengthening exercises to support the knee joint and improve overall function. 5. Patient Response:    - Individualized Approach: Tailor the initiation and progression of ROM exercises based on the patient’s individual response. If the patient experiences increased pain or instability with ROM exercises, adjustments may be needed, and they should be guided by a healthcare professional. 6. Physical Therapy:    - Professional Guidance: In many cases, physical therapy is recommended as part of non-operative management. A physical therapist can design a specific exercise program and monitor progress, ensuring that exercises are introduced and advanced at the appropriate times based on the patient’s condition.   Summary: Start ROM exercises when the acute symptoms have subsided and the knee is stable enough to safely perform these movements. Initially, focus on gentle exercises and progressively increase intensity as tolerated. Professional guidance from a physical therapist can help ensure exercises are performed correctly and effectively, minimizing the risk of exacerbating instability? |
| **What are the indications for nonoperative management for patients with patellar instability?** | Nonoperative management for patients with patellar instability is often indicated based on several factors. These include the severity of the instability, the patient’s functional needs, and the response to initial treatments. Here are the primary indications for opting for nonoperative management:   1. Mild to Moderate Instability:    - Single or Infrequent Dislocations: If the patient has experienced a limited number of dislocations or subluxations, nonoperative management may be effective.    - Lack of Severe Structural Damage: When there is no significant underlying bony or soft tissue damage that requires surgical intervention. 2. Acute Management and Recovery:    - Initial Phase Post-Injury: Nonoperative treatment is often the first line of management immediately following a dislocation or acute episode to reduce pain, swelling, and inflammation. 3. Response to Conservative Measures:    - Effectiveness of Physical Therapy: If the patient responds well to physical therapy, including strengthening exercises for the quadriceps and other supporting muscles, and improves their stability and function.    - Pain Management: If symptoms can be managed effectively with medications, braces, and activity modification. 4. Functional Improvement:    - Return to Activity: When nonoperative measures lead to satisfactory improvement in knee function, allowing the patient to return to their daily activities without significant symptoms or instability. 5. Patient Preferences and Lifestyle:    - Conservative Approach Preference: If the patient prefers to avoid surgery and is willing to adhere to a conservative treatment plan.    - Lifestyle Considerations: For individuals whose lifestyle or occupation does not demand high-impact activities or sports, nonoperative management might be sufficient. 6. Presence of Contributing Factors:    - Correctable Factors: When instability is primarily due to modifiable factors such as muscle weakness, poor alignment, or improper biomechanics, and these factors can be addressed through physical therapy and rehabilitation. 7. Preoperative Trial:    - Pre-Surgical Trial: Sometimes, nonoperative management is used as a trial period before considering surgery. This allows for assessment of the patient's response to conservative measures and can help guide the decision on whether surgery is necessary. 8. Young or Active Individuals:    - Young Age with Mild Symptoms: In younger patients with mild symptoms or occasional instability, conservative management might be preferred to avoid the potential risks and long-term outcomes associated with surgery.   Summary: Nonoperative management is indicated for patients with patellar instability who have mild to moderate symptoms, respond well to conservative treatments, do not have significant structural damage, and prefer a conservative approach. It includes physical therapy, pain management, activity modification, and possibly bracing. The decision should be individualized based on the patient’s specific situation and needs. |
| **What are the contraindications for nonoperative management for patients with patellar instability** | Nonoperative management for patellar instability may not be suitable in certain situations where conservative measures are less likely to be effective or safe. Contraindications for nonoperative management include:   1. Severe Structural Damage:    - Significant Cartilage Damage: Extensive damage to the articular cartilage or osteochondral lesions that may not be adequately managed with nonoperative methods.    - Bone Fractures: Significant bony abnormalities or fractures around the patella or femur that require surgical intervention. 2. Frequent and Severe Dislocations:    - Recurrent Dislocations: Patients with frequent, severe, or multiple episodes of patellar dislocation or subluxation that do not improve with conservative measures may need surgical intervention. 3. Persistent Symptoms Despite Conservative Treatment:    - Ineffective Conservative Measures: If symptoms, including pain and instability, persist despite a full course of conservative treatment, including physical therapy, bracing, and activity modification. 4. Associated Ligament Injuries:    - Significant Ligament Damage: Presence of significant damage to key stabilizing structures like the medial patellofemoral ligament (MPFL) or other knee ligaments that may require surgical repair. 5. Malalignment or Anatomical Abnormalities:    - Severe Patellar or Trochlear Dysplasia: Significant bony malalignment, such as severe patellar or trochlear dysplasia, that cannot be effectively addressed through nonoperative measures alone. 6. Functional Limitations:    - Significant Functional Impairment: Patients who experience substantial limitations in daily activities or who have a high level of functional demand that cannot be met through conservative management. 7. Patient Factors and Preferences:    - Patient's Expectations and Preferences: If the patient desires a more definitive solution or has specific functional goals that are unlikely to be achieved with nonoperative management. 8. Inadequate Compliance:    - Poor Adherence to Conservative Treatment: If the patient is unable or unwilling to adhere to the prescribed conservative treatment plan, including physical therapy, exercise regimens, and lifestyle modifications. 9. Infection or Systemic Issues:    - Underlying Health Issues: Presence of systemic health problems or infections that complicate conservative management or increase the risk of poor outcomes.   Summary: Nonoperative management is contraindicated when there is severe structural damage, persistent symptoms despite conservative treatment, significant ligament injuries, severe anatomical abnormalities, substantial functional impairment, patient preference for more definitive solutions, poor adherence to treatment, or underlying health issues that complicate the management. In such cases, surgical options may be more appropriate to address the underlying issues and provide better long-term outcomes. |
| **What are the indications for MPFL reconstruction for patients with patellar instability?** | Medial Patellofemoral Ligament (MPFL) reconstruction is often considered for patients with patellar instability when nonoperative treatments have not provided sufficient relief or when there are specific indications that suggest a surgical approach might be necessary. The primary indications for MPFL reconstruction include:   1. Recurrent Patellar Dislocations or Subluxations:    - Frequent Episodes: Patients with multiple dislocations or subluxations of the patella that significantly impact their quality of life and daily function, despite conservative treatment. 2. Failed Nonoperative Management:    - Ineffective Conservative Treatment: When conservative measures, such as physical therapy, bracing, and activity modification, have not effectively controlled symptoms or improved stability. 3. Documented MPFL Injury:    - Severe MPFL Injury: Evidence of significant injury or rupture of the MPFL on imaging studies (MRI or arthroscopy) that is contributing to patellar instability. 4. Severe Patellar Malalignment:    - Patellar Malalignment: Significant patellar malalignment, including conditions like patellar alta (high-riding patella) or trochlear dysplasia, that cannot be managed effectively with conservative treatment alone. 5. Associated Structural Abnormalities:    - Underlying Bony Abnormalities: Presence of severe anatomical abnormalities, such as abnormal patellar tracking or significant trochlear dysplasia, that are contributing to instability. 6. High Functional Demand:    - Athletic or High-Demand Activities: Patients who are involved in high-impact sports or activities and who experience instability that limits their ability to participate in these activities. 7. Significant Pain and Functional Limitations:    - Impact on Daily Life: Patients who experience significant pain, functional limitations, or disability due to patellar instability, and where surgical intervention may offer a better long-term outcome. 8. Previous Surgical Interventions:    - Failed Previous Surgeries: In cases where prior surgical interventions aimed at addressing patellar instability have failed, MPFL reconstruction might be considered as a secondary option. 9. Patient Preferences:    - Desire for Surgical Intervention: Patients who prefer a definitive surgical solution after understanding the potential risks and benefits and who are willing to undergo the procedure.   Summary: MPFL reconstruction is indicated for patients with recurrent patellar dislocations or subluxations, failed conservative management, significant MPFL injury, severe patellar malalignment, associated bony abnormalities, high functional demands, significant pain and limitations, or who have previously failed other surgical interventions. The decision to proceed with MPFL reconstruction should be individualized, taking into account the patient’s specific condition, functional needs, and overall health. |
| **Are there any different considerations that should be made in pediatric patients undergoing MPFL reconstruction for patellar instability?** | Yes, there are several important considerations when performing MPFL (Medial Patellofemoral Ligament) reconstruction in pediatric patients with patellar instability. These considerations differ from those for adults due to the ongoing growth and development of the pediatric skeleton. Key considerations include:   1. Growth Plate Preservation:    - Avoiding Injury to Growth Plates: Care must be taken to avoid damage to the growth plates (epiphyseal plates) during surgery. Surgeons often use techniques and fixation methods that minimize the risk of affecting growth plate development.    - Techniques and Implants: The choice of surgical techniques and implants may be adjusted to avoid interfering with bone growth. For instance, some surgeons use bioabsorbable or non-permanent fixation devices to avoid long-term impact on growth. 2. Growth Spurts and Development:    - Timing of Surgery: The timing of surgery can be critical. It is important to consider the patient's growth spurts and developmental stages. Surgery might be timed to coincide with periods of slower growth or planned around major growth spurts. 3. Anatomical Considerations:    - Anatomical Differences: Pediatric patients have different anatomical characteristics compared to adults, including smaller bone structures and differences in patellar and femoral anatomy. These factors must be considered when planning and executing the surgery.    - Patellar Development: The development of the patella and its alignment can differ in children. The surgeon must account for these variations to ensure proper patellar tracking and alignment post-surgery. 4. Rehabilitation and Recovery:    - Rehabilitation Protocols: Rehabilitation protocols may need to be adjusted for pediatric patients. These protocols should account for the patient’s age, activity level, and the need for gentle, age-appropriate rehabilitation exercises.    - Growth and Healing: The healing process in pediatric patients can differ from adults due to ongoing growth and bone remodeling. Rehabilitation must be tailored to accommodate these factors. 5. Long-Term Outcomes:    - Long-Term Follow-Up: Pediatric patients will require long-term follow-up to monitor the success of the surgery, the integrity of the MPFL reconstruction, and any effects on growth and development.    - Revisions and Adjustments: There may be a need for revision surgery or adjustments in the future, particularly if the patient experiences further growth or develops new issues related to patellar instability. 6. Psychosocial Factors:    - Patient and Family Education: It is important to educate both the patient and their family about the nature of the surgery, expected outcomes, and the importance of adhering to postoperative care and rehabilitation. 7. Activity and Sports Participation:    - Return to Sports: Considerations about the timing of return to sports and high-impact activities should be made carefully to ensure the patient’s safety and the success of the surgery.   Summary: MPFL reconstruction in pediatric patients requires careful consideration of growth plate preservation, anatomical differences, timing of surgery, and rehabilitation protocols. Long-term follow-up and adjustments may be needed to account for ongoing growth and development. A tailored approach to both the surgical technique and postoperative care is essential for optimal outcomes in pediatric patients. |
| **What clinical factors may influence your decision to perform a TTO for patellar instability?** | A Tibial Tuberosity Osteotomy (TTO) is a surgical procedure often considered for patients with patellar instability when other nonoperative or less invasive surgical treatments are insufficient. The decision to perform a TTO depends on several clinical factors:   1. Severity and Frequency of Patellar Instability:    - Recurrent Dislocations/Subluxations: Patients who experience frequent or severe patellar dislocations or subluxations that are not manageable through conservative treatment or MPFL reconstruction alone may benefit from TTO.    - Chronic Instability: Chronic or long-standing instability that affects the patient’s daily activities and quality of life. 2. Patellar Malalignment:    - Patellar Maltracking: Significant patellar maltracking or misalignment, such as patellar alta (high-riding patella) or patellar tilt, that contributes to instability.    - Trochlear Dysplasia: Presence of severe trochlear dysplasia that affects patellar tracking and requires realignment of the tibial tuberosity to improve patellar stability. 3. Failure of Conservative and Other Surgical Treatments:    - Ineffectiveness of Nonoperative Management: When conservative treatments, including physical therapy and bracing, have not been successful.    - Insufficient Results from MPFL Reconstruction: If MPFL reconstruction alone has not resolved the patellar instability or if there is ongoing patellar malalignment that MPFL reconstruction cannot fully address. 4. Anatomical Considerations:    - High Tibial Tuberosity (TT) Position: A high tibial tuberosity, which affects patellar tracking and stability, may indicate the need for a TTO to correct the alignment.    - Patellar Bony Anatomy: Consideration of the patient’s bony anatomy, including the position of the tibial tuberosity relative to the patella and femur. 5. Functional Demands:    - Athletic or High-Demand Activities: Patients who participate in high-impact sports or activities where precise patellar alignment is critical for performance and function. 6. Patient Age and Growth:    - Age Appropriateness: For younger patients, especially those with ongoing growth, careful consideration is needed to avoid interference with growth plates. The timing of surgery should be planned to minimize impact on bone development. 7. Associated Pathologies:    - Presence of Additional Injuries: If there are associated injuries or conditions, such as cartilage damage or ligament injuries, that require comprehensive management. 8. Patient and Family Preferences:    - Patient Goals and Expectations: The patient’s goals, expectations, and willingness to undergo a more invasive procedure like TTO should be considered. Discussion with the patient and their family about the potential benefits and risks is essential. 9. Postoperative Rehabilitation and Compliance:    - Ability to Adhere to Rehabilitation: The patient’s ability to adhere to postoperative rehabilitation protocols is important, as TTO requires a period of immobilization and physical therapy for optimal recovery.   Summary: The decision to perform a Tibial Tuberosity Osteotomy (TTO) for patellar instability is influenced by the severity and frequency of instability, presence of patellar malalignment, failure of other treatments, anatomical considerations, functional demands, patient age, associated pathologies, patient preferences, and ability to comply with rehabilitation. TTO is typically considered when these factors suggest that realigning the tibial tuberosity will provide significant benefits in improving patellar stability and overall function. |
| **Which factor(s), if any, should be considered when deciding which trochleoplasty technique to perform?** | When deciding on a trochleoplasty technique for managing patellar instability, several factors should be carefully considered to choose the most appropriate method for the patient. Here are key factors to take into account:   1. Degree and Type of Trochlear Dysplasia:    - Severity of Dysplasia: The severity of trochlear dysplasia, as classified by imaging studies (e.g., MRI, CT scans) or grading systems (e.g., Dejour classification), influences the choice of technique. Severe dysplasia may require more extensive reconstruction.    - Type of Dysplasia: The specific type of dysplasia (e.g., flat, dysplastic, or hypoplastic trochlea) and associated patellar tracking issues will guide the selection of the appropriate technique. 2. Patient’s Age and Growth Considerations:    - Age: In younger patients, particularly those with ongoing growth, techniques that minimize interference with growth plates and future bone development are preferred.    - Growth Plates: Techniques that avoid disrupting growth plates are crucial for pediatric and adolescent patients. 3. Anatomical and Structural Considerations:    - Patellar Position and Alignment: The patellar alignment and position in relation to the femur must be considered. Techniques that best address the specific malalignment or instability observed in the patient will be chosen.    - Bone Quality and Anatomy: The quality of the underlying bone and the anatomical characteristics of the femur and trochlea influence technique selection. 4. Associated Pathologies:    - Other Joint Pathologies: The presence of associated conditions, such as cartilage damage or other ligamentous injuries, may impact the choice of trochleoplasty technique or necessitate a combined approach. 5. Technical Expertise and Experience:    - Surgeon’s Experience: The surgeon's familiarity and expertise with specific trochleoplasty techniques play a significant role. Choosing a technique that aligns with the surgeon’s skills and experience can improve outcomes. 6. Functional and Performance Goals:    - Patient’s Activity Level: The patient’s activity level and functional demands, including participation in sports or high-impact activities, will influence the choice of technique to ensure optimal outcomes. 7. Potential Complications and Risks:    - Risk of Complications: Different techniques have varying risks and potential complications. Consideration of these risks, such as infection, implant failure, or residual instability, is important in decision-making. 8. Rehabilitation and Recovery:    - Postoperative Rehabilitation: The expected postoperative rehabilitation process and the ability of the patient to adhere to the rehabilitation protocol should be considered. Some techniques may require more intensive or prolonged rehabilitation.   Summary: Choosing a trochleoplasty technique involves evaluating the severity and type of trochlear dysplasia, patient age, anatomical factors, associated pathologies, surgeon expertise, patient functional goals, potential complications, and the rehabilitation process. Each factor contributes to selecting the technique that will best address the patient’s specific needs and provide the most favorable outcomes. |

**Table 2.** Perplexity AI

| **Question** | **Answer** |
| --- | --- |
| **What factors of patient history should be evaluated in the setting of patellar instability** | When evaluating a patient with patellar instability, several key factors of the patient history should be assessed:   1. Number and timing of dislocation events:Whether this is a first-time dislocation or recurrent instability 2. Mechanism of injury:Whether it was traumatic or non-contact 3. Symptoms:Pain levels and location 4. Prior treatments:Whether reduction was required 5. Symptoms between instability episodes:Presence of pain or instability during daily activities or sports 6. Family history:Any family members with patellar instability or hypermobility 7. Activity level and sports participation:Type and intensity of physical activities 8. Associated conditions:History of contralateral patellar dislocation 9. Impact on daily life:Limitations in activities or sports participation   Obtaining a detailed history on these factors helps guide diagnosis, assess risk of recurrence, and inform treatment decisions for patients with patellar instability. The clinician should pay particular attention to differentiating between pain and instability as the primary complaint, as this impacts management strategies. |
| **What aspects of the physical exam should be performed in patients with patellar instability** | Based on the search results and clinical guidelines, several key aspects of the physical exam should be performed in patients with suspected patellar instability:   1. Inspection:  - Assess for knee effusion, which is common in acute patellar dislocations - Evaluate overall lower limb alignment, looking for factors like genu valgum/varum  1. Palpation:  - Check for tenderness over the medial retinaculum, which may indicate a torn medial patellofemoral ligament (MPFL) - Palpate the superior, inferior, medial and lateral aspects of the patella  1. Range of motion and tracking:  - Assess patellar mobility and glide, particularly medial and lateral patellar glide - Look for a J-sign, which indicates lateral patellar maltracking during knee extension  1. Special tests:  - Perform the lateral patellar apprehension test, considered one of the best tests for patellar instability - Consider the gravity subluxation test and medial apprehension test, especially in patients with prior surgery  1. Muscle strength and timing:  - Evaluate quadriceps strength, particularly the vastus medialis obliquus (VMO) - Assess for any delay in VMO contraction compared to vastus lateralis  1. Associated structures:  - Examine hip range of motion and strength - Perform a full knee ligament exam  1. Gait analysis:  - Observe the patient's gait pattern   The examiner should also assess for factors that may contribute to instability, such as generalized ligamentous laxity, femoral anteversion, and tibial torsion. It's important to compare findings to the contralateral knee when possible. While some tests like the Q-angle have been traditionally used, recent studies suggest they may have limited reliability in this patient population |
| **When should advanced imaging (MRI/CT) be performed in patients presenting with patellar instability?** | Based on the search results, advanced imaging like MRI or CT should be performed in the following situations for patients presenting with patellar instability:   1. After the initial injury or dislocation event: MRI is considered critical for any patient who has sustained a patellar dislocation, whether it's their first time or a recurrence. It helps identify:Bone marrow edema pattern on the medial patella and lateral trochlea 2. To assess anatomical risk factors: MRI and CT are useful for evaluating key predisposing factors for instability, including:Trochlear dysplasia 3. When surgical management is being considered: Advanced imaging helps guide surgical decision-making. It's particularly important for:Detecting intra-articular free bodies 4. To evaluate rotational deformities: CT can be useful to assess rotational abnormalities like femoral anteversion or excessive tibial external rotation when severe bony malalignment is suspected. 5. For comprehensive assessment: Cross-sectional imaging provides a more complete evaluation of patellofemoral joint morphology compared to plain radiographs alone.   While CT has traditionally been used, there is an increasing trend towards using MRI for comprehensive evaluation of patellar instability. MRI offers the advantage of assessing cartilaginous surfaces and detecting associated soft tissue injuries without radiation exposure |
| **When should patients start range of motion exercises when undergoing non-operative management for patellar instability?** | Based on the available information, here are some guidelines for when patients should start range of motion exercises during non-operative management of patellar instability:   1. Initial phase:  - In the acute phase immediately following a patellar dislocation, the focus is typically on reducing pain and swelling. During this time, patients are often immobilized in a knee brace or splint.  1. Early motion:  - Gentle range of motion exercises can usually begin within the first 1-2 weeks after injury, as tolerated by the patient. - The initial focus is on regaining full knee extension and gradually increasing flexion.  1. Progressive mobilization:  - As pain and swelling decrease, typically around 2-4 weeks post-injury, more active range of motion exercises can be introduced. - This may include exercises like heel slides and stationary cycling with minimal resistance.  1. Full motion:  - The goal is to achieve full range of motion by 6-8 weeks post-injury in most cases.  1. Individualized approach:  - The exact timing should be tailored to each patient based on factors like pain levels, swelling, and tissue healing. - Patients with recurrent instability may need a more conservative approach compared to first-time dislocations.   It's important to note that range of motion exercises should be performed under the guidance of a physical therapist or physician to ensure proper technique and prevent re-injury. The progression of exercises should be gradual and pain-free to optimize recovery and reduce the risk of recurrent instability. |
| **What are the indications for nonoperative management for patients with patellar instability?** | Based on the search results, the main indications for nonoperative management of patellar instability are:   1. First-time patellar dislocation without an osteochondral fracture: Nonoperative treatment is recommended as the first-line approach for patients experiencing their first patellar dislocation, provided there is no associated osteochondral fracture. 2. Acute patellar dislocations: The initial treatment for acute patellar dislocations is often nonoperative, consisting of physical therapy and immobilization techniques. 3. Patients without significant anatomical risk factors: Those without major predisposing factors like severe trochlear dysplasia or patella alta may be good candidates for nonoperative management. 4. Mild to moderate instability: Patients with less severe instability symptoms that do not significantly impact daily activities may be managed nonoperatively initially. 5. Trial period before considering surgery: Nonoperative treatment is typically attempted for 3 to 6 months before surgical options are considered. 6. Patient preference: Some patients may prefer to try conservative management before considering surgical interventions. 7. Contraindications to surgery: Patients who are poor surgical candidates due to medical comorbidities or other factors may be managed nonoperatively.   It's important to note that while nonoperative management is often the first-line treatment, the outcomes can be variable. A systematic review found that patient-reported outcomes consistently improved after nonoperative treatment, but did not return to normal, and redislocation rates remained high. Therefore, careful monitoring and reassessment are necessary during nonoperative management to determine if surgical intervention becomes indicated. |
| **What are the contraindications for nonoperative management for patients with patellar instability** | Based on the search results and clinical guidelines, the main contraindications for nonoperative management of patellar instability include:   1. Osteochondral fracture: The presence of an associated osteochondral fracture is a key contraindication for nonoperative management. These fractures often require surgical intervention. 2. Substantial disruption of the medial patellofemoral ligament (MPFL): Significant damage to the MPFL and medial soft tissue structures may indicate a need for surgical repair or reconstruction. 3. Recurrent or chronic instability: Patients with a history of recurrent dislocations or chronic instability are often candidates for surgical intervention rather than continued nonoperative management. 4. Severe anatomical risk factors: The presence of significant predisposing factors such as severe trochlear dysplasia, patella alta, or excessive tibial tubercle-trochlear groove (TT-TG) distance may necessitate surgical correction. 5. Failed conservative management: If a patient has undergone an appropriate course of nonoperative treatment (typically 3-6 months) without improvement, surgical options may be considered. 6. High-demand athletes: Competitive athletes or those participating in high-risk activities may benefit from earlier surgical intervention to facilitate a quicker return to sport and reduce the risk of recurrence. 7. Persistent functional limitations: If patellar instability significantly impacts a patient's daily activities or quality of life despite conservative measures, surgery may be indicated. 8. Large chondral injuries: The presence of substantial cartilage damage may require surgical management to address both the instability and the chondral lesion.   It's important to note that the decision between operative and nonoperative management should be individualized based on the patient's specific circumstances, including age, activity level, and personal goals. The presence of these contraindications does not always mandate surgery, but they are factors that may influence the treatment decision towards operative management. |
| **What are the indications for MPFL reconstruction for patients with patellar instability?** | Based on the search results, the main indications for MPFL (medial patellofemoral ligament) reconstruction in patients with patellar instability include:   1. Recurrent patellar instability:Multiple patellar dislocations (typically 2 or more) 2. Failed conservative treatment:Persistent instability symptoms after a course of nonoperative management 3. Primary patellar dislocation with specific factors:Ongoing instability symptoms 4. Normal or near-normal anatomy:Normal tibial tuberosity-trochlear groove (TT-TG) distance (≤20 mm) 5. Painful subluxation with:Excessive lateral mobility/laxity on examination 6. Imaging findings:Positive CT or MRI findings consistent with patellar instability   It's important to note that MPFL reconstruction is most effective when there are no significant bony abnormalities contributing to instability. In cases with severe trochlear dysplasia, significant patella alta, or excessive TT-TG distance, additional procedures may be necessary in conjunction with or instead of isolated MPFL reconstruction. |
| **Are there any different considerations that should be made in pediatric patients undergoing MPFL reconstruction for patellar instability?** | Yes, there are several important considerations specific to pediatric patients undergoing MPFL reconstruction for patellar instability:   1. Physeal considerations:The proximity of the femoral attachment site to the distal femoral physis is a key concern in skeletally immature patients. 2. Graft fixation techniques:Modified techniques may be used to minimize risk to the physis, such as: 3. Graft choice:While autografts are commonly used, some surgeons prefer allografts in pediatric patients to avoid donor site morbidity and preserve autograft options for future procedures. 4. Growth considerations:There is potential for the reconstructed MPFL to become too tight as the child grows, which may necessitate revision surgery. 5. Skeletal maturity assessment:Careful evaluation of skeletal maturity is crucial for surgical planning and technique selection. 6. Associated anatomical factors:Pediatric patients may have ongoing development of predisposing factors like trochlear dysplasia, which can influence surgical decision-making. 7. Rehabilitation protocols:May need to be modified based on the patient's age and skeletal maturity. 8. Long-term outcomes:The long-term effects of MPFL reconstruction in growing patients are still being studied, necessitating careful follow-up. 9. Potential for revision:There may be a higher likelihood of needing revision surgery in the future due to growth or development of other instability factors. 10. Concomitant procedures:The need for additional procedures (e.g., tibial tubercle osteotomy) is generally avoided in skeletally immature patients.   These considerations highlight the importance of tailoring the surgical approach to the specific needs and developmental stage of pediatric patients with patellar instability. |
| **What clinical factors may influence your decision to perform a TTO for patellar instability?** | Based on the search results and clinical guidelines, several key clinical factors may influence the decision to perform a tibial tubercle osteotomy (TTO) for patellar instability:   1. Tibial tubercle-trochlear groove (TT-TG) distance:A TT-TG distance ≥20 mm is often considered an indication for TTO. 2. Patella alta:The presence of patella alta, often measured by the Caton-Deschamps index (CDI), may indicate a need for TTO. 3. Trochlear dysplasia:The presence and severity of trochlear dysplasia may influence the decision for TTO. 4. Recurrent instability:Patients with a history of multiple patellar dislocations or failed conservative treatment may be candidates for TTO. 5. Skeletal maturity:TTO is generally avoided in skeletally immature patients to prevent growth disturbances. 6. Cartilage damage:The presence of significant cartilage damage or osteochondral lesions may influence the decision for TTO. 7. Failure of isolated soft tissue procedures:If a patient has failed previous isolated soft tissue procedures (e.g., MPFL reconstruction), TTO may be considered. 8. Coronal plane malalignment:Some surgeons use the tibial tubercle-posterior cruciate ligament (TT-PCL) distance as an alternative measure, with values >20 mm considered pathologic. 9. Activity level and goals:The patient's activity level, especially participation in high-risk sports, may influence the decision for TTO. 10. Combined procedures:TTO is often performed in combination with other procedures like MPFL reconstruction and lateral retinacular release, based on the specific patient factors.   It's important to note that the decision to perform TTO should be individualized based on the patient's specific anatomical and clinical factors, as well as their functional goals. A comprehensive evaluation, including imaging studies and physical examination, is crucial in determining the most appropriate treatment approach. |
| **Which factor(s), if any, should be considered when deciding which trochleoplasty technique to perform?** | Based on the search results, several factors should be considered when deciding which trochleoplasty technique to perform for patients with patellar instability:   1. Grade of trochlear dysplasia:The severity and type of trochlear dysplasia influences the choice of technique. High-grade dysplasia may require more extensive procedures like sulcus deepening trochleoplasty. 2. Quality of cartilage and subchondral bone:In young patients with good quality cartilage and subchondral bone, a thin-flap technique may be preferred as it allows for a more malleable flap. 3. Extent of the trochlear bump:The depth and length of the trochleoplasty can be titrated according to the extent of the trochlear bump, particularly with thin-flap techniques. 4. Presence of patellofemoral osteoarthritis:Pre-existing patellofemoral osteoarthritis may influence the choice of technique and the potential risks/benefits of the procedure. 5. Patient age and skeletal maturity:This factor is important for considering the risk of growth disturbances and the potential need for future revisions. 6. Associated anatomical factors:The presence of other contributing factors like patella alta or increased tibial tubercle-trochlear groove (TT-TG) distance may influence the decision to combine trochleoplasty with other procedures. 7. Surgeon experience and preference:Different techniques (e.g., lateral facet elevation, sulcus deepening, recession wedge) have varying levels of technical difficulty and may be chosen based on surgeon familiarity and expertise. 8. Goals of the procedure:Whether the primary goal is to address instability, improve patellofemoral articulation, or potentially alter the natural history of patellofemoral degeneration. 9. Need for concomitant procedures:The decision may be influenced by whether additional procedures like MPFL reconstruction or lateral retinacular lengthening are planned. 10. Long-term considerations:The potential impact on future development of osteoarthritis and the possibility of needing revision surgery should be taken into account.   These factors highlight the importance of individualizing the surgical approach based on patient-specific characteristics and the surgeon's assessment of the best technique to address the underlying pathology while minimizing risks. |

**Table 3.** Bing CoPilot

| **Question** | **Answer** |
| --- | --- |
| **What factors of patient history should be evaluated in the setting of patellar instability** | When evaluating a patient with patellar instability, several factors from their history should be considered:   1. Demographic Information: Age, gender, and body mass index can influence the risk and management of patellar instability. 2. Activity Level: Current and past activity levels, including participation in sports, can provide insight into the potential causes and future risks. 3. Mechanism of Injury: Understanding how the injury occurred, such as a direct blow or a twisting motion, is crucial 4. Previous Injuries: A history of previous knee injuries, especially prior patellar dislocations, is a significant risk factor. 5. Anatomical Factors: Conditions like trochlear dysplasia, patella alta, genu valgum, and ligamentous laxity should be assessed 6. Skeletal Maturity: Younger patients with open growth plates may have different treatment considerations 7. Family History: A family history of similar issues can indicate a genetic predisposition.   These factors help in forming a comprehensive understanding of the patient’s condition and tailoring the treatment plan accordingly. Is there a specific aspect of patellar instability you’re particularly interested in? |
| **What aspects of the physical exam should be performed in patients with patellar instability** | When examining a patient with patellar instability, several key aspects of the physical exam should be performed:   1. Inspection: Look for signs of swelling, bruising, or deformity around the knee. Note any abnormal patellar positioning or tracking. 2. Palpation: Check for tenderness, especially along the medial patellofemoral ligament (MPFL) and the patellar tendon. Feel for any crepitus or grinding sensations. 3. Range of Motion: Assess the knee’s range of motion to identify any limitations or pain during movement. 4. Patellar Mobility Tests:    - Apprehension Test: Gently push the patella laterally while the knee is flexed to 30 degrees. A positive test is indicated by the patient’s apprehension or attempt to contract the quadriceps to prevent dislocation.    - Patellar Tilt Test: Evaluate the lateral tilt of the patella by lifting the lateral edge. A positive test indicates excessive lateral tilt.    - Quadrant Test: Measure the passive translation of the patella in medial and lateral directions, comparing it to the contralateral side. 5. Dynamic Assessment: Observe the patellar tracking during active knee flexion and extension. Look for signs like the J sign, where the patella moves laterally as the knee extends. 6. Ligamentous Laxity Tests: Assess for generalized ligamentous laxity, which can contribute to patellar instability.   These components help in diagnosing the extent and nature of patellar instability, guiding appropriate treatment plans. Is there a specific test or aspect you’re curious about? |
| **When should advanced imaging (MRI/CT) be performed in patients presenting with patellar instability?** | Advanced imaging, such as MRI or CT, should be considered in patients with patellar instability under the following circumstances:   1. Acute Injury: If there is suspicion of osteochondral injuries, which are associated with poor outcomes if not addressed. 2. Recurrent Instability: In cases of recurrent patellar dislocations to assess underlying anatomical abnormalities like trochlear dysplasia, patella alta, or excessive tibial tubercle–trochlear groove distance. 3. Pre-Surgical Planning: When planning surgical interventions, advanced imaging helps in detailed anatomical assessment and surgical planning. 4. Persistent Symptoms: If the patient continues to experience symptoms despite conservative treatment, advanced imaging can help identify subtle pathologies.   These imaging modalities provide detailed views of the bone and soft tissue structures, aiding in accurate diagnosis and effective treatment planning. Is there a specific aspect of imaging you’re curious about? |
| **When should patients start range of motion exercises when undergoing non-operative management for patellar instability?** | Patients undergoing non-operative management for patellar instability should start range of motion (ROM) exercises as soon as comfort permits1. This typically means beginning with gentle, pain-free movements to avoid further injury and to promote healing.  Here are some general guidelines:   1. Initial Phase (1-3 weeks): Focus on reducing swelling and pain. Gentle passive and active-assisted ROM exercises can be started as tolerated. 2. Intermediate Phase (3-6 weeks): Gradually increase ROM exercises, aiming to restore full knee extension and flexion. 3. Advanced Phase (6+ weeks): Continue to progress ROM exercises and begin incorporating strengthening exercises once full ROM is achieved and the patient can perform them without apprehension.   It’s important to tailor the rehabilitation program to the individual patient’s progress and comfort levels. Always follow the specific recommendations of the treating physician or physical therapist.  Is there a particular exercise or phase you’re interested in learning more about? |
| **What are the indications for nonoperative management for patients with patellar instability?** | Nonoperative management for patellar instability is typically indicated in the following scenarios:   1. First-Time Dislocation: Patients experiencing their first patellar dislocation without associated osteochondral fractures or loose bodies. 2. Minimal Symptoms: Patients with mild symptoms who do not experience frequent dislocations or significant functional impairment. 3. No Significant Anatomical Abnormalities: When imaging does not reveal severe anatomical abnormalities like trochlear dysplasia or patella alta. 4. Patient Preference: Some patients may prefer nonoperative management due to personal or medical reasons.   Nonoperative treatment often includes bracing, physical therapy, and activity modification to strengthen the muscles around the knee and improve patellar tracking.  Is there a specific aspect of nonoperative management you’re interested in exploring further? |
| **What are the contraindications for nonoperative management for patients with patellar instability** | Nonoperative management for patellar instability is generally contraindicated in the following scenarios:   1. Osteochondral Lesions: Presence of osteochondral fractures or loose bodies that require surgical intervention. 2. Recurrent Instability: Patients with frequent dislocations or chronic instability that significantly impacts their daily activities. 3. Severe Anatomical Abnormalities: Conditions such as severe trochlear dysplasia, patella alta, or a significantly increased tibial tubercle–trochlear groove distance. 4. Medial Patellofemoral Ligament (MPFL) Tear: A complete tear of the MPFL, especially on the femoral side. 5. Systemic or Local Infection: Presence of infection that could complicate conservative treatment. 6. Poor Bone Condition: Conditions like rheumatoid arthritis that affect bone quality.   These factors often necessitate surgical intervention to properly address the underlying issues and prevent further complications. Is there a specific contraindication you would like more information on? |
| **What are the indications for MPFL reconstruction for patients with patellar instability?** | Medial Patellofemoral Ligament (MPFL) reconstruction is indicated for patients with patellar instability under the following conditions:   1. Recurrent Patellar Instability: Patients experiencing frequent dislocations or subluxations despite conservative treatment. 2. Failure of Nonoperative Management: When nonoperative measures, such as physical therapy and bracing, have not successfully stabilized the patella. 3. Associated Anatomical Abnormalities: Presence of anatomical factors like trochlear dysplasia, patella alta, or increased tibial tubercle–trochlear groove distance that contribute to instability. 4. Acute Injury with MPFL Tear: Acute patellar dislocation resulting in a significant tear or avulsion of the MPFL. 5. Unstable Osteochondral Fractures: Presence of osteochondral fractures or loose bodies that require surgical intervention.   MPFL reconstruction aims to restore stability to the patella by reconstructing the ligament that helps prevent lateral dislocation. Is there a specific aspect of the procedure you’re interested in learning more about? |
| **Are there any different considerations that should be made in pediatric patients undergoing MPFL reconstruction for patellar instability?** | Yes, there are several important considerations for pediatric patients undergoing MPFL reconstruction for patellar instability:   1. Skeletal Maturity: Pediatric patients often have open growth plates, so it’s crucial to avoid damaging these areas during surgery. Techniques like all-epiphyseal drilling are used to prevent growth disturbances. 2. Graft Selection: The choice of graft is important. Autografts, such as the gracilis tendon, are commonly used, but allografts are gaining popularity due to their ability to preserve native tissue and their superior mechanical properties. 3. Anatomical Considerations: Pediatric patients may have different anatomical challenges, such as trochlear dysplasia or patella alta, which need to be addressed during surgery. 4. Postoperative Care: Rehabilitation protocols may need to be adjusted for younger patients to ensure they are age-appropriate and consider the patient’s growth and development. 5. Psychosocial Factors: Younger patients may require additional support to understand and comply with postoperative care and rehabilitation. Engaging parents and caregivers in the process is essential.   These considerations help ensure that the surgical and postoperative management is tailored to the unique needs of pediatric patients, promoting better outcomes and minimizing complications. Is there a specific aspect of pediatric MPFL reconstruction you’re curious about? |
| **What clinical factors may influence your decision to perform a TTO for patellar instability?** | The decision to perform a Tibial Tubercle Osteotomy (TTO) for patellar instability is influenced by several clinical factors:   1. Tibial Tubercle–Trochlear Groove (TT-TG) Distance: A TT-TG distance greater than 20 mm is a common indication for TTO, as it suggests significant lateralization of the tibial tubercle. 2. Patella Alta: This condition, where the patella sits higher than normal, can contribute to instability and may necessitate TTO to improve patellar tracking. 3. Trochlear Dysplasia: Severe trochlear dysplasia, where the trochlear groove is shallow or flat, can be a complicating factor. TTO can help by realigning the patella to better engage with the trochlear groove. 4. Recurrent Instability: Patients with recurrent patellar dislocations or subluxations that have not responded to conservative treatments may benefit from TTO. 5. Failed Previous Surgeries: If previous surgical interventions have failed to stabilize the patella, TTO may be considered as a corrective measure. 6. Combined Procedures: TTO is often performed in conjunction with other procedures, such as MPFL reconstruction, to address multiple contributing factors to patellar instability.   These factors help determine the appropriateness of TTO in providing a more stable and functional knee. Is there a specific aspect of TTO you’re interested in learning more about? |
| **Which factor(s), if any, should be considered when deciding which trochleoplasty technique to perform?** | When deciding which trochleoplasty technique to perform for patellar instability, several factors should be considered:   1. Severity of Trochlear Dysplasia: The type and severity of trochlear dysplasia (e.g., Dejour types B and D) can influence the choice of technique. Deepening trochleoplasty is often used for severe dysplasia, while recession wedge trochleoplasty may be preferred for less severe cases. 2. Patient’s Age and Skeletal Maturity: Younger patients with open growth plates may require techniques that minimize the risk of growth disturbances. 3. Associated Anatomical Abnormalities: Presence of conditions like patella alta, increased Q angle, or lateralized tibial tuberosity may necessitate combining trochleoplasty with other procedures. 4. Surgeon’s Experience and Preference: The surgeon’s familiarity and comfort with specific techniques can also play a role in the decision-making process3. 5. Patient’s Activity Level and Expectations: Active patients or athletes may benefit from techniques that provide more robust stabilization, while less active patients may require less invasive options. 6. Postoperative Rehabilitation Considerations: The anticipated rehabilitation process and potential for complications, such as postoperative stiffness, should be factored into the decision.   These considerations help tailor the surgical approach to the individual patient’s needs, ensuring the best possible outcomes. Is there a specific technique or factor you’re particularly interested in? |

**Table 4.** Claude2

| **Question** | **Answer** |
| --- | --- |
| **What factors of patient history should be evaluated in the setting of patellar instability** | When evaluating a patient with patellar instability, several key factors in the patient's history should be assessed. These include:   1. Age and gender: Patellar instability is more common in adolescents and young adults, particularly females. 2. Number and circumstances of dislocation events:    - First-time vs. recurrent dislocations    - Traumatic vs. atraumatic dislocations 3. Mechanism of injury: Direct impact, twisting motion, or spontaneous dislocation 4. Sports and activity level: Type and intensity of physical activities or sports involvement 5. Family history of patellar instability or hypermobility syndromes 6. Previous treatments and their outcomes:    - Conservative management attempts    - Prior surgeries 7. Symptoms:    - Pain location and characteristics    - Feelings of instability or "giving way"    - Locking or catching sensations 8. Functional limitations: Impact on daily activities, work, or sports performance 9. History of other knee injuries or conditions 10. Developmental history: Any childhood lower limb alignment issues or gait abnormalities 11. General medical history: Connective tissue disorders, inflammatory conditions, or other systemic diseases that might affect joint stability 12. Growth and skeletal maturity (in younger patients) 13. Presence of generalized ligamentous laxity 14. History of contralateral knee problems or patellar instability   This comprehensive history helps in understanding the nature and severity of the patellar instability, guiding further physical examination, imaging studies, and ultimately, the treatment plan.  Would you like more information on how any of these factors specifically influence the management of patellar instability? |
| **What aspects of the physical exam should be performed in patients with patellar instability** | The physical examination for patients with patellar instability should be comprehensive and include several key elements. Here are the important aspects to assess:   1. Gait analysis:    - Observe for any abnormalities in walking pattern 2. Lower limb alignment:    - Assess for genu valgum or varum    - Evaluate for rotational deformities 3. Q-angle measurement:    - Typically increased in patients with patellar instability 4. Patellar tracking:    - Observe patellar movement during active knee flexion and extension (J-sign) 5. Patellar apprehension test:    - Apply lateral pressure on the patella while extending the knee 6. Patellar mobility:    - Assess medial and lateral patellar translation 7. Knee range of motion:    - Both active and passive 8. Quadriceps strength and function:    - Look for atrophy or weakness 9. Ligament stability tests:    - Assess integrity of cruciate and collateral ligaments 10. Generalized ligamentous laxity:     - Beighton score assessment 11. Palpation:     - Check for tenderness along medial retinaculum and MPFL 12. Assessment of hip strength and function:     - Particularly hip abductors and external rotators 13. Foot and ankle examination:     - Look for pes planus or excessive pronation 14. Neurovascular examination of the lower limb 15. Assessment of core strength and balance 16. Evaluation of patellar height:     - Insall-Salvati ratio (can be confirmed with imaging) 17. Evaluation of femoral anteversion and tibial torsion 18. Examination of the contralateral knee for comparison   This thorough physical examination, combined with the patient's history and appropriate imaging studies, helps in diagnosing patellar instability, identifying contributing factors, and guiding treatment decisions.  Would you like more details on how to perform any of these specific examination techniques? |
| **When should advanced imaging (MRI/CT) be performed in patients presenting with patellar instability?** | Advanced imaging such as MRI (Magnetic Resonance Imaging) or CT (Computed Tomography) should be considered in specific situations for patients presenting with patellar instability. Here are the key indications:   1. Suspected osteochondral injury:    - When there's concern for cartilage damage or loose bodies in the joint 2. Recurrent instability:    - To evaluate for predisposing anatomic factors and assess the extent of soft tissue damage 3. Surgical planning:    - When surgery is being considered, to guide the choice of procedure 4. Unclear diagnosis:    - When clinical examination and plain radiographs are inconclusive 5. Assessment of MPFL (Medial Patellofemoral Ligament) injury:    - MRI can help evaluate the extent of MPFL damage 6. Trochlear dysplasia evaluation:    - CT or MRI can provide detailed information about trochlear morphology 7. Tibial tubercle-trochlear groove (TT-TG) distance measurement:    - CT is particularly useful for this assessment 8. Post-traumatic cases:    - To rule out associated injuries like meniscal tears or other ligament injuries 9. Chronic cases:    - To assess for degenerative changes or long-term complications 10. Pediatric patients:     - When physeal status needs to be evaluated for surgical planning 11. Patellar or trochlear bone marrow edema:     - MRI can detect this sign of recent dislocation or subluxation 12. Evaluation of patellar tilt and engagement:     - Dynamic MRI studies can be helpful in some cases   It is important to note that while advanced imaging can provide valuable information, it should not replace a thorough clinical examination and basic radiographics. The decision to perform advanced imaging should be based on how it will impact the management plan for the individual patient. Would you like more information on the specific findings these imaging modalities can reveal in patellar instability cases. |
| **When should patients start range of motion exercises when undergoing non-operative management for patellar instability?** | In non-operative management of patellar instability, the timing of range of motion (ROM) exercises is crucial for optimal recovery. Here's a general guideline:   1. Acute phase (0-2 weeks post-dislocation):    - Typically, immediate ROM exercises are encouraged within pain limits    - Focus on gentle, passive ROM to prevent stiffness    - Active assisted exercises may begin, avoiding extremes of flexion 2. Sub-acute phase (2-6 weeks):    - Progress to more active ROM exercises    - Gradually increase flexion range as tolerated 3. Recovery phase (6+ weeks):    - Full active ROM exercises   Key considerations:   - Individual variations: The exact timing may vary based on the severity of the injury, associated soft tissue damage, and the patient's pain levels. - Pain-guided progression: Exercises should be performed within pain limits. - Avoid provocative positions: Initially limit activities that put the patella at risk of redislocation (e.g., deep squats). - Concurrent therapies: ROM exercises are often combined with quadriceps strengthening and neuromuscular training. - Supervision: Initial exercises are best performed under the guidance of a physical therapist. - Bracing: Some protocols may use a brace to limit ROM initially, gradually increasing allowed motion.   The goal is to balance the need for early motion to prevent stiffness and promote healing, with the need to protect healing tissues and prevent re-injury. Close monitoring and individualization of the protocol are essential for optimal outcomes. |
| **What are the indications for nonoperative management for patients with patellar instability?** | **5. What are the indications for nonoperative management for patients with patellar instability?**  Nonoperative management is often the first-line treatment for many patients with patellar instability. The indications for nonoperative management include:   1. First-time (primary) patellar dislocation:    - In the absence of significant osteochondral injury or complete MPFL tear 2. Minimal anatomical risk factors:    - Patients without significant trochlear dysplasia, patella alta, or increased TT-TG distance 3. Acute phase after dislocation:    - Initial management to control pain and swelling 4. Patients with low physical demands:    - Those who don't participate in high-risk activities or competitive sports 5. Skeletally immature patients:    - To avoid potential growth disturbances from surgical intervention 6. Mild symptoms of instability:    - Patients experiencing minor or infrequent episodes of instability 7. Good response to physical therapy:    - Patients showing improvement with conservative measures 8. Patient preference:    - Those who wish to avoid surgery and accept the risk of recurrence 9. Medical contraindications to surgery:    - Patients with comorbidities that increase surgical risks 10. Absence of significant functional limitation:     - Patients able to perform daily activities without major issues 11. No evidence of progressive cartilage damage:     - When imaging shows no significant articular surface injury 12. Presence of generalized ligamentous laxity:     - As these patients may benefit from focused physical therapy and proprioception training 13. Patients willing to modify activities:     - Those able to avoid high-risk movements or sports 14. Cases of atraumatic subluxation without full dislocation   Nonoperative management typically involves a combination of physical therapy, activity modification, bracing or taping techniques, and patient education. The goal is to improve quadriceps strength, enhance patellar stability, and reduce the risk of recurrent instability.  It's important to note that while nonoperative management is often successful, patients should be monitored for recurrence of instability or development of complications that might necessitate surgical intervention.  Would you like more information on specific nonoperative treatment strategies or their outcomes? |
| **What are the contraindications for nonoperative management for patients with patellar instability** | While nonoperative management is often the first-line treatment for patellar instability, there are certain situations where it may be contraindicated or less likely to be successful. The contraindications or relative contraindications for nonoperative management include:   1. Large osteochondral fragments:    - Presence of substantial loose bodies that require removal 2. Complete tear of the medial patellofemoral ligament (MPFL):    - Especially if associated with significant displacement 3. Recurrent dislocations:    - Multiple episodes of patellar dislocation, indicating failure of conservative measures 4. Significant anatomical risk factors:    - Severe trochlear dysplasia    - High-grade patella alta    - Significantly increased tibial tubercle-trochlear groove (TT-TG) distance 5. Persistent functional instability:    - Despite adequate trial of physical therapy and bracing 6. High-demand athletes:    - Particularly those involved in pivoting and cutting sports who require a quicker return to play 7. Presence of significant cartilage damage:    - Progressive articular cartilage injury that may worsen without surgical intervention 8. Failed previous nonoperative management:    - Lack of improvement after a thorough course of conservative treatment 9. Concomitant injuries requiring surgery:    - Such as associated ligament or meniscal injuries 10. Chronic instability with muscle atrophy:     - Significant quadriceps weakness that hasn't responded to physical therapy 11. Patellar tendon rupture or quadriceps tendon rupture:     - Associated with the dislocation event 12. Skeletally mature patients with clear indications for realignment:     - Such as those with significant malalignment that won't correct without surgery 13. Occupational requirements:     - Jobs that demand high knee stability and can't accommodate activity modification 14. Patient preference for definitive treatment:     - After understanding the risks and benefits of surgical vs. nonoperative management 15. Locked knee due to interposed soft tissues or osteochondral fragments   It's important to note that these contraindications are not absolute in all cases, and the decision between operative and nonoperative management should be made on an individual basis, considering factors such as the patient's age, activity level, specific anatomical factors, and personal goals.  Would you like more information on how these contraindications influence the decision-making process for treatment? |
| **What are the indications for MPFL reconstruction for patients with patellar instability?** | MPFL (Medial Patellofemoral Ligament) reconstruction is a common surgical procedure for patellar instability. The indications for MPFL reconstruction include:   1. Recurrent patellar dislocations:    - Multiple episodes of dislocation despite conservative management 2. Failed conservative treatment:    - Persistent instability after adequate nonoperative management 3. Confirmed MPFL tear or incompetence:    - Diagnosed through clinical examination and imaging studies 4. Normal to mild trochlear dysplasia:    - MPFL reconstruction alone may be sufficient in these cases 5. Normal alignment and patellar height:    - When other anatomical factors are not significantly contributing to instability 6. High-demand athletes:    - Especially those involved in pivoting and cutting sports 7. Chronic instability with apprehension:    - Patients with persistent fear of dislocation affecting daily activities 8. Absence of significant patellofemoral arthritis:    - As advanced arthritis might require different treatment approaches 9. Skeletally mature patients:    - To avoid potential growth disturbances in pediatric patients 10. Anatomic risk factors that don't require bony procedures:     - When tibial tubercle transfer or trochleoplasty are not necessary 11. Traumatic patellar dislocation with MPFL rupture:     - Especially in cases with persistent instability 12. Complementary procedure:     - In combination with other stabilizing procedures when indicated 13. Patellar maltracking without significant bony malalignment:     - When soft tissue imbalance is the primary cause of instability 14. Patient preference:     - After thorough discussion of risks and benefits 15. Occupational requirements:     - Jobs demanding high knee stability that can't be achieved with conservative measures 16. Absence of generalized ligamentous laxity:     - As isolated MPFL reconstruction may be less effective in these cases 17. Normal to slightly increased Q-angle:     - When significant correction of alignment is not necessary   It's important to note that the decision to perform MPFL reconstruction should be based on a comprehensive evaluation of the patient's specific anatomical factors, activity level, and goals. In some cases, MPFL reconstruction may be combined with other procedures to address multiple factors contributing to patellar instability.  Would you like more information on the surgical technique or outcomes of MPFL reconstruction? |
| **Are there any different considerations that should be made in pediatric patients undergoing MPFL reconstruction for patellar instability?** | Yes, there are several important considerations specific to pediatric patients undergoing MPFL reconstruction for patellar instability:   1. Skeletal maturity:    - Careful evaluation of growth plates is crucial    - Avoid procedures that may damage or cross the physis 2. Graft choice and fixation:    - Autografts (often hamstring) are preferred over allografts    - Soft tissue fixation methods are often used to avoid drilling through the physis 3. Surgical technique modification:    - Femoral attachment may need to be proximal to the physis    - Consider physeal-sparing techniques 4. Growth potential:    - Account for future growth and its impact on graft tension and placement 5. Ligament laxity:    - Pediatric patients often have more generalized laxity, which may affect outcomes 6. Compliance with rehabilitation:    - May be more challenging in younger patients 7. Long-term outcomes:    - Consider the longevity of the reconstruction through growth periods 8. Associated anatomical factors:    - Assess for developmental issues like trochlear dysplasia or patella alta 9. Activity level and sports participation:    - Balance the need for stability with allowing normal childhood activities 10. Timing of surgery:     - Consider delaying until closer to skeletal maturity if possible 11. Risk of recurrence:     - Generally higher in pediatric populations 12. Psychological factors:     - Address fear of re-injury and impact on sports participation 13. Family involvement:     - Crucial for decision-making and post-operative care 14. Alternative treatments:     - Consider other options like bracing or activity modification more extensively 15. Growth disturbances:     - Monitor for potential issues like leg length discrepancy post-surgery 16. Rehabilitation protocols:     - May need to be modified for age and compliance ability 17. Concurrent procedures:     - Carefully consider the necessity and timing of additional stabilizing procedures 18. Long-term follow-up:     - More extensive monitoring through growth periods is necessary   These considerations aim to balance the need for patellar stability with the preservation of normal growth and development in pediatric patients. The approach often requires a more conservative and carefully planned strategy compared to adult patients.  Would you like more information on any specific aspect of managing patellar instability in pediatric patients? |
| **What clinical factors may influence your decision to perform a TTO for patellar instability?** | The decision to perform a tibial tubercle osteotomy (TTO) for patellar instability is influenced by several clinical factors. Here are the key considerations:   1. Increased TT-TG (Tibial Tubercle-Trochlear Groove) distance:    - Generally, a TT-TG distance >20mm is considered an indication for TTO 2. Patella alta:    - Elevated Caton-Deschamps index (>1.2) or Insall-Salvati ratio (>1.3) 3. Patellar tilt:    - Significant lateral patellar tilt on imaging studies 4. Q-angle:    - Increased Q-angle contributing to lateral vector forces on the patella 5. Failed previous soft tissue procedures:    - Recurrent instability after MPFL reconstruction or other soft tissue surgeries 6. Trochlear dysplasia:    - Presence of significant trochlear dysplasia, especially high-grade 7. Chondral damage:    - Presence of lateral facet chondral lesions that might benefit from unloading 8. Skeletal maturity:    - TTO is generally reserved for skeletally mature patients 9. Recurrent instability:    - Multiple episodes of patellar dislocation despite conservative management 10. Alignment issues:     - Valgus malalignment or excessive femoral anteversion 11. Activity level:     - High-demand athletes or patients with occupational requirements for stability 12. Associated patellofemoral pain:     - Especially in cases where anteriorization might help with pain relief 13. Body habitus:     - Consideration in patients with high BMI where soft tissue procedures alone may be insufficient 14. Patellar tracking:     - Significant lateral maltracking during knee flexion and extension 15. Age:     - Generally more suitable for younger adults, with caution in older patients due to increased risk of nonunion 16. Ligamentous laxity:     - Consideration in patients with generalized ligamentous laxity where bony procedures may be more beneficial 17. Presence of open growth plates:     - TTO is contraindicated in skeletally immature patients 18. Concomitant procedures:     - Often performed in combination with MPFL reconstruction for comprehensive stabilization   It's important to note that the decision to perform a TTO should be based on a comprehensive evaluation of these factors, along with careful consideration of the patient's individual needs and goals. The specific type of TTO (medialization, anteriorization, or distalization) would be determined based on the predominant contributing factors to the instability.  Would you like more information on the different types of TTO or the surgical technique? |
| **Which factor(s), if any, should be considered when deciding which trochleoplasty technique to perform?** | When deciding on a trochleoplasty technique for patellar instability, several factors should be considered. The choice of technique depends on the specific anatomical abnormalities and the severity of trochlear dysplasia. Here are the key factors to consider:   1. Dejour classification of trochlear dysplasia:    - Type A: Shallow trochlea    - Type B: Flat or convex trochlea    - Type C: Hypoplastic medial facet    - Type D: Cliff pattern with convexity 2. Sulcus angle:    - The degree of flatness or convexity of the trochlea 3. Lateral trochlear facet height:    - Particularly important in determining the need for deepening trochleoplasty 4. Presence of supratrochlear spur:    - May influence the choice between recession or deepening techniques 5. Patellar height:    - Patella alta may influence the extent and type of trochlear correction needed 6. Cartilage quality:    - The condition of the articular cartilage can affect the feasibility of certain techniques 7. Patient age:    - Younger patients may benefit from less aggressive approaches 8. Associated pathologies:    - Presence of chondral lesions or osteoarthritis 9. Patellar shape and size:    - To ensure congruence after trochlear reshaping 10. Severity of instability:     - More severe cases may require more extensive remodeling 11. Surgeon experience and preference:     - Familiarity with specific techniques can influence choice 12. Need for concomitant procedures:     - Such as MPFL reconstruction or tibial tubercle osteotomy 13. Skeletal maturity:     - Generally reserved for skeletally mature patients 14. Extent of lateral femoral condyle prominence:     - May influence the choice of lateral facet elevation techniques 15. Presence of crossing sign on lateral radiographs:     - Indicative of the need for trochlear groove deepening   Common trochleoplasty techniques include:   1. Deepening trochleoplasty (Bereiter technique):    - For severe dysplasia with a flat or convex trochlea 2. Lateral facet elevation:    - When the lateral facet is hypoplastic 3. Recession trochleoplasty:    - For cases with a prominent supratrochlear spur 4. Groove deepening:    - For milder cases of dysplasia 5. Thin-flap (Dejour) technique:    - A variation of deepening trochleoplasty   The choice of technique should be tailored to the individual patient's anatomy and the specific goals of the surgery. It's crucial to carefully evaluate all these factors to select the most appropriate trochleoplasty technique for optimal outcomes.  Would you like more detailed information on any specific trochleoplasty technique or its indications? |

**Table 5.** Google Gemini

| **Question** | **Answers** |
| --- | --- |
| **What factors of patient history should be evaluated in the setting of patellar instability** | When evaluating a patient with patellar instability, it's essential to gather a comprehensive history to understand the underlying causes and risk factors. Here are key factors to consider:  1. History of Instability:   - Frequency: How often has the patella dislocated or subluxated? - Triggering events: What activities or movements typically lead to instability? - Reducing maneuvers: Does the patient have any techniques to manually reduce the patella?   2. Pain and Symptoms:   - Pain location: Where does the pain occur? Is it in the knee, thigh, or other areas? - Intensity: How severe is the pain? Does it interfere with daily activities? - Locking: Does the knee "lock" or "give out" during or after an episode of instability?   3. Previous Injuries:   - Knee injuries: Have there been any previous injuries to the knee, such as ACL tears, MCL tears, or meniscus damage? - Other injuries: Any other injuries, especially those involving the hip or ankle, could contribute to patellar instability.   4. Medical History:   - Systemic diseases: Are there any underlying medical conditions that could affect joint stability, such as hypermobility syndromes or metabolic disorders? - Medications: Are there any medications that might influence joint function or muscle strength?   5. Family History:   - Patellar instability: Is there a family history of patellar instability or other joint problems?   6. Activities and Lifestyle:   - Sports and hobbies: What activities does the patient engage in? Are they high-impact or twisting sports? - Occupation: Does the patient's job involve repetitive knee movements or heavy lifting? - Fitness level: How physically active is the patient?   7. Anatomical Factors:   - Knee alignment: Is there evidence of genu valgum (knock-knees) or genu varum (bowlegs)? - Q angle: Has the Q angle (the angle between the quadriceps tendon and the patellar tendon) been measured? A larger Q angle can increase the risk of patellar instability   By carefully considering these factors, healthcare providers can gain valuable insights into the potential causes of patellar instability and develop an appropriate treatment plan. |
| **What aspects of the physical exam should be performed in patients with patellar instability** | A thorough physical examination is essential for diagnosing patellar instability and identifying any contributing factors.Here are key aspects to be evaluated:  1. Inspection:   - Alignment: Observe the alignment of the knee, looking for signs of genu valgum (knock-knees) or genu varum (bowlegs). - Swelling: Check for any swelling or tenderness around the knee joint. - Patellar position: Assess the position of the patella relative to the femoral groove.   2. Palpation:   - Tenderness: Palpate the knee joint for any areas of tenderness, especially around the patella and the femoral groove. - Crepitus: Check for any grating or popping sounds during knee movement.   3. Range of Motion:   - Flexion and extension: Assess the full range of motion of the knee joint. - Patellar mobility: Evaluate the mobility of the patella by gently moving it medially and laterally.   4. Stability Tests:   - Apprehension test: This test is used to assess the likelihood of patellar subluxation or dislocation. The patient is placed supine, and the examiner gently pushes the patella laterally while the knee is extended. - Pivot shift test: This test is used to evaluate the stability of the anterior cruciate ligament (ACL). It involves flexing the knee, externally rotating the tibia, and then applying a valgus stress.   5. Muscle Strength:   - Quadriceps strength: Assess the strength of the quadriceps muscles, as they play a crucial role in stabilizing the patella. - Hamstring strength: Evaluate the strength of the hamstring muscles, as they can also influence patellar stability.   6. Functional Testing:   - Squatting: Observe the patient's squatting technique to assess patellar tracking and any signs of instability. - Running or jumping: If the patient is involved in sports, assess their ability to perform activities that may trigger patellar instability.   7. Special Tests:   - Patella grind test: This test is used to assess for patellofemoral pain. The patient is asked to flex and extend the knee while the examiner applies pressure on the patella.   By carefully evaluating these aspects during the physical examination, healthcare providers can gain a better understanding of the underlying causes of patellar instability and develop an appropriate treatment plan. |
| **When should advanced imaging (MRI/CT) be performed in patients presenting with patellar instability?** | Advanced imaging, such as MRI or CT, is typically recommended in patients with patellar instability when:   - Initial physical examination and history are inconclusive: If the physical examination and history do not clearly identify the underlying cause of patellar instability, imaging can help to visualize the structures involved. - There are signs of significant pain or dysfunction: If the patient is experiencing severe pain, locking, or significant limitations in daily activities, imaging can help to identify any structural abnormalities or injuries that may be contributing to these symptoms. - There is a suspicion of a concomitant injury: If there is a concern that a patellar instability may be associated with another injury, such as an ACL tear or meniscus damage, imaging can help to confirm or rule out these possibilities. - Non-operative treatment has failed: If conservative measures, such as physical therapy, bracing, or medication,have not been effective in managing the patellar instability, imaging can help to identify any underlying structural issues that may require surgical intervention.   MRI is generally preferred over CT for imaging patellar instability due to its superior soft tissue contrast, which allows for better visualization of structures like cartilage, tendons, and ligaments. However, CT may be considered in certain situations, such as when there is a suspicion of a fracture or bone abnormality.  It's important to note that the decision to order advanced imaging should be made on a case-by-case basis, considering the patient's individual symptoms, history, and physical examination findings. |
| **When should patients start range of motion exercises when undergoing non-operative management for patellar instability?** | The timing of starting range of motion exercises for patients undergoing non-operative management for patellar instability depends on the severity of the condition and the individual patient's response to treatment.  Generally, gentle range of motion exercises can be started shortly after the acute phase of the injury has subsided.This typically involves reducing pain and inflammation. Once the initial pain and swelling have improved, gradual progression of range of motion exercises can begin.  However, it's important to consult with a healthcare professional or physical therapist to determine the appropriate timing and intensity of exercises. They can assess the patient's condition and provide personalized guidance based on their specific needs.  Factors that may influence the timing of starting range of motion exercises include:   - Severity of the injury: More severe cases of patellar instability may require a longer period of rest before starting exercises. - Pain and swelling: The presence of significant pain or swelling may limit the ability to perform range of motion exercises. - Individual patient factors: Factors such as age, overall fitness level, and previous injuries can also influence the timing and progression of exercises.   It's generally recommended to start with gentle, pain-free exercises and gradually increase the range of motion and intensity over time. This approach can help to prevent re-injury and promote optimal healing. |
| **What are the indications for nonoperative management for patients with patellar instability?** | Nonoperative management is often considered for patients with patellar instability when:   - The condition is mild or moderate: Patients with mild or moderate symptoms may benefit from nonoperative treatment, especially if there are no significant underlying structural abnormalities. - There are no significant underlying structural abnormalities: If the patellar instability is not caused by a severe structural issue, such as a large patella or a deep femoral groove, nonoperative management may be effective. - The patient is young and physically active: Younger patients who are involved in sports or other physical activities may be more likely to benefit from nonoperative treatment, as they have a higher potential for recovery and rehabilitation. - The patient is willing to comply with the treatment plan: Nonoperative management often requires a significant commitment from the patient, including physical therapy, bracing, and lifestyle modifications. If the patient is not willing or able to adhere to the treatment plan, surgical intervention may be more appropriate.   Common nonoperative treatment approaches for patellar instability include:   - Physical therapy: This involves exercises to strengthen the quadriceps muscles, improve flexibility, and correct any biomechanical imbalances. - Bracing: Braces can help to support the patella and prevent it from dislocating. - Lifestyle modifications: Patients may need to avoid activities that can exacerbate patellar instability, such as running, jumping, or squatting. - Medication: In some cases, pain relievers or anti-inflammatory medications may be prescribed to help manage symptoms.   While nonoperative management can be effective for many patients with patellar instability, it's important to note that it may not be suitable for everyone. If nonoperative treatment fails to improve symptoms or if the condition is severe,surgical intervention may be necessary. |
| **What are the contraindications for nonoperative management for patients with patellar instability** | Contraindications for nonoperative management of patellar instability include:   - Severe patellar instability: If the patella frequently dislocates or subluxates, despite nonoperative treatment,surgical intervention may be necessary to stabilize the joint. - Significant structural abnormalities: If there are severe structural abnormalities, such as a large patella, a shallow femoral groove, or a tight lateral retinaculum, nonoperative treatment may be less likely to be effective. - Persistent pain and dysfunction: If the patient continues to experience significant pain or limitations in daily activities despite nonoperative treatment, surgical intervention may be considered. - Failed nonoperative treatment: If previous attempts at nonoperative management have been unsuccessful,surgical intervention may be necessary to address the underlying cause of the patellar instability.   It's important to note that these contraindications may vary depending on the individual patient's circumstances and the severity of their patellar instability. A healthcare professional can evaluate the patient's condition and determine the most appropriate course of treatment. |
| **What are the indications for MPFL reconstruction for patients with patellar instability?** | MPFL (Medial Patellofemoral Ligament) reconstruction is often considered for patients with patellar instability when:   - Nonoperative treatment has failed: If conservative measures, such as physical therapy, bracing, and medication,have not been successful in managing patellar instability, MPFL reconstruction may be an option. - There is a clear history of patellar subluxation or dislocation: If the patient has experienced repeated episodes of the patella moving out of place, MPFL reconstruction can help to stabilize the joint. - There is a documented MPFL tear or insufficiency: If imaging studies, such as MRI, reveal a tear or insufficiency of the MPFL, reconstruction can be considered. - The patient is young and physically active: Younger patients who are involved in sports or other physical activities may be more likely to benefit from MPFL reconstruction, as they have a higher potential for recovery and return to function. - There are no other significant contraindications: The patient should not have any other conditions that would make MPFL reconstruction risky or ineffective.   It's important to note that MPFL reconstruction is a surgical procedure with potential risks and complications.The decision to undergo this surgery should be made in consultation with a qualified orthopedic surgeon who can assess the patient's individual circumstances and determine if the benefits outweigh the risks. |
| **Are there any different considerations that should be made in pediatric patients undergoing MPFL reconstruction for patellar instability?** | Yes, there are several considerations that should be made in pediatric patients undergoing MPFL reconstruction for patellar instability:   - Growth potential: Pediatric patients are still growing, and the MPFL and other structures around the knee may continue to develop. This must be taken into account when planning the surgery and choosing the appropriate graft material. - Bone maturity: The bones of pediatric patients may not be fully mature, which can affect the healing process and the long-term outcome of the surgery. - Activity level: Pediatric patients are often more physically active than adults, which can increase the risk of re-injury after MPFL reconstruction. - Psychological factors: Children and adolescents may have different psychological concerns and coping mechanisms than adults, which should be considered during the rehabilitation process.   Specific considerations for pediatric MPFL reconstruction may include:   - Graft choice: Autologous grafts, such as hamstring tendons or quadriceps tendons, are often preferred in pediatric patients due to their growth potential. - Surgical technique: The surgical technique may need to be modified to accommodate the smaller size of pediatric structures. - Rehabilitation: The rehabilitation program for pediatric patients should be tailored to their age, activity level, and growth potential. - Long-term follow-up: Pediatric patients who undergo MPFL reconstruction may require longer-term follow-up to monitor their growth and development and to assess the long-term outcome of the surgery.   It's important to consult with a pediatric orthopedic surgeon who has experience with MPFL reconstruction in children to discuss the specific considerations and risks associated with this procedure. |
| **What clinical factors may influence your decision to perform a TTO for patellar instability?** | Clinical Factors Influencing TTO for Patellar Instability  TTO (Tibial Tuberosity Osteotomy) is a surgical procedure used to realign the patella and improve its tracking in the femoral groove. The decision to perform a TTO is influenced by several clinical factors:  1. Patellar Anatomy:   - Patella alta: A high-riding patella can increase the risk of subluxation or dislocation. - Patella magna: A large patella can also contribute to instability. - Q angle: A large Q angle (the angle between the quadriceps tendon and the patellar tendon) can increase the risk of patellar instability.   2. Previous Injuries:   - ACL tears: If the patient has a history of ACL tears, it may increase the risk of patellar instability and influence the decision to perform a TTO. - Meniscus tears: Chronic meniscus tears can contribute to patellar instability and may require addressing in conjunction with a TTO.   3. Patient Factors:   - Age: Younger patients may be more likely to benefit from TTO due to their potential for recovery and return to function. - Activity level: Patients who are involved in high-impact sports or activities may have a higher risk of patellar instability and may benefit from TTO. - Patient preference: The patient's willingness to undergo surgery and their expectations for the procedure should be considered.   4. Failed Nonoperative Treatment:   - Physical therapy: If physical therapy, bracing, or other nonoperative measures have not been effective in managing patellar instability, TTO may be considered.   5. Other Considerations:   - Risk of re-injury: The risk of re-injury after TTO should be weighed against the potential benefits. - Potential complications: The patient should be informed of the potential complications of TTO, such as infection,nerve damage, and patellar tendon rupture.   It's important to note that the decision to perform a TTO should be made on a case-by-case basis, considering the individual patient's circumstances and the potential risks and benefits of the procedure. A qualified orthopedic surgeon can evaluate the patient's condition and provide personalized guidance. |
| **Which factor(s), if any, should be considered when deciding which trochleoplasty technique to perform?** | The choice of trochleoplasty technique for patellar instability depends on several factors:  1. Severity of Trochlear Abnormality:   - Degree of dysplasia: The severity of trochlear dysplasia, as determined by imaging studies, is a key factor in selecting the appropriate technique. Mild dysplasia may require a less invasive approach, while severe dysplasia may necessitate a more extensive procedure.   2. Patient Factors:   - Age: Younger patients may be more suitable for certain techniques that involve bone grafting or cartilage transplantation. - Activity level: The patient's level of physical activity can influence the choice of technique. More active patients may require a procedure that provides greater stability. - Previous surgeries: Any previous surgeries in the knee joint, such as ACL reconstruction or meniscus repair, may influence the choice of trochleoplasty technique.   3. Surgeon Experience:   - Technique expertise: The surgeon's experience and preference for a particular trochleoplasty technique can also be a factor. Some surgeons may have more experience with certain techniques than others.   4. Specific Trochleoplasty Techniques:   - Trochlear deepening: This technique involves deepening the trochlear groove to improve patellar tracking. It may be suitable for patients with mild to moderate trochlear dysplasia. - Trochlear resurfacing: This technique involves resurfacing the trochlear groove with cartilage or a cartilage substitute. It may be appropriate for patients with more severe trochlear dysplasia. - Trochlear osteotomy: This technique involves realigning the trochlear groove to improve patellar tracking. It may be considered for patients with severe trochlear dysplasia or combined with other procedures.   5. Combined Procedures:   - Simultaneous procedures: In some cases, trochleoplasty may be combined with other procedures, such as MPFL reconstruction or patellar tendon lengthening, to address multiple factors contributing to patellar instability.   Ultimately, the best trochleoplasty technique for a given patient will depend on a careful evaluation of these factors and the surgeon's expertise. A thorough preoperative assessment and discussion with the surgeon can help patients make informed decisions about their treatment options. |
